# Novel Genomic Regions Linked to *Ascochyta* Blight Resistance in Two Differentially Resistant Cultivars of Chickpea

**DOI:** 10.3389/fpls.2022.762002

**Published:** 2022-04-25

**Authors:** Fida Alo, Anupalli Roja Rani, Michael Baum, Sarvjeet Singh, Zakaria Kehel, Upasana Rani, Sripada Udupa, Khaled Al-Sham’aa, Alsamman M. Alsamman, Tawffiq Istanbuli, Basem Attar, Aladdin Hamwieh, Ahmed Amri

**Affiliations:** ^1^International Center for Agricultural Research in the Dry Areas (ICARDA), Beirut, Lebanon; ^2^Department of Genetics and Biotechnology, Osmania University, Hyderabad, India; ^3^Department of Plant Breeding and Genetics, Punjab Agricultural University, Ludhiana, India; ^4^African Genome Center, Mohammed VI Polytechnic University, Ben Guerir, Morocco; ^5^Agriculture Genetic Engineering Research Institute, Giza, Egypt; ^6^The Scottish Association for Marine Science, Scottish Marine Institute, Oban, United Kingdom

**Keywords:** *Ascochyta* blight, chickpea, genotyping-by-sequencing (GBS), quantitative trait loci (QTLs), resistance

## Abstract

*Ascochyta* blight (AB), caused by the fungal pathogen *Ascochyta rabiei*, is a devastating foliar disease of chickpea (*Cicer arietinum* L.). The genotyping-by-sequencing (GBS)-based approach was deployed for mapping QTLs associated with AB resistance in chickpea in two recombinant inbred line populations derived from two crosses (AB_3279_ derived from ILC 1929 × ILC 3279 and AB_482_ derived from ILC 1929 × ILC 482) and tested in six different environments. Twenty-one different genomic regions linked to AB resistance were identified in regions CalG02 and CalG04 in both populations AB_3279_ and AB_482_. These regions contain 1,118 SNPs significantly associated with AB resistance (*p* ≤ 0.001), which explained 11.2–39.3% of the phenotypic variation (PVE). Nine of the AB resistance-associated genomic regions were newly detected in this study, while twelve regions were known from previous AB studies. The proposed physical map narrows down AB resistance to consistent genomic regions identified across different environments. Gene ontology (GO) assigned these QTLs to 319 genes, many of which were associated with stress and disease resistance, and with most important genes belonging to resistance gene families such as leucine-rich repeat (LRR) and transcription factor families. Our results indicate that the flowering-associated gene GIGANTEA is a possible key factor in AB resistance in chickpea. The results have identified AB resistance-associated regions on the physical genetic map of chickpea and allowed for the identification of associated markers that will help in breeding of AB-resistant varieties.

## Introduction

Chickpea (*Cicer arietinum* L.) is the second most important grain legume cultivated in arid and semi-arid regions of the world. Chickpea originated from Turkey and Syria, and is presently grown in more than 57 countries under varied environmental conditions (Merga and Haji, 2019). Globally, chickpea production is 11.67 million tons, and chickpea is cultivated in over 14 million hectares of land (FAO, 2019). Chickpea is consumed by ∼22% of the world’s population as a primary source of protein (Wettberg et al., 2018).

Chickpea is a self-pollinating diploid (2*n* = 16) annual crop with a genome size of 738 Mbp (Varshney et al., 2013). Chickpea grows under diverse ecological conditions; therefore, its production and yield performance can be severely affected by abiotic and biotic stresses (Gaur et al., 2012). Among biotic stresses, *Ascochyta* blight (AB), caused by the fungus *Ascochyta rabiei* (Pass.) Labr (Sagi et al., 2017), teleomorph *Didymella rabiei*, is responsible for significant losses in chickpea yield and quality (Armstrong-Cho et al., 2008). There is a high level of genetic variability and divergence among *D. rabiei* populations in almost all chickpea-growing regions of the world (Chongo et al., 2004), which presents a major challenge for chickpea breeders as resistance to AB is not durable, because of the high variability of *A. rabiei* populations (Rakshit et al., 2003).

Complete resistance or high level of resistance to *D. rabiei* has not been found in chickpea cultivars so far, and the resistance shown by many cultivars is either partial or incomplete (Jayakumar et al., 2005). Some studies support the idea of oligogenic inheritance, where resistance is conferred by one or two genes (Bhardwaj et al., 2010), while others support the idea that AB resistance is conferred by polygenic inheritance, and several quantitative trait loci (QTLs) have been identified in different mapping populations (Sabbavarapu et al., 2013; Stephens et al., 2014; Garg et al., 2018; Kushwah et al., 2021).

Recent advances in DNA sequencing technology have provided effective tools for sequence-based single nucleotide polymorphism (SNP) marker discovery and genotyping. Genotyping-by-sequencing (GBS) can be performed to discover thousands of markers in almost any genome in a population (Poland and Rife, 2012). GBS can be used to develop high-density linkage maps and QTL mapping in various crops including chickpea. SNP markers obtained from GBS have been used in chickpea to identify QTLs controlling seed traits (Verma et al., 2015), early flowering trait, and resistance to AB (Gaur et al., 2015; Daba et al., 2016; Deokar et al., 2019; Kushwah et al., 2021).

Recently, Li et al. (2017) identified a 100-kb genomic region containing 12 candidate genes for *Ascochyta* resistance associated with a major QTL on chromosome 4 of chickpea using genome-wide association mapping (GWAS) approaches. Subsequently, Sagi et al. (2017) identified 121 NBS-LRR genes and studied their structural diversity and important role in *Ascochyta* resistance (Liu et al., 2012; Takagi et al., 2013). Deokar et al. (2018) identified eleven QTLs related to AB resistance. Additionally, three next-generation sequencing (NGS) approaches have been used to understand the complex mechanisms of AB resistance in chickpea, using diverse chickpea genotypes where some differentially expressed genes (DEGs) and miRNAs were detected as a response to AB infection (Garg et al., 2019).

Developing chickpea varieties with durable resistance is considered the most effective and sustainable strategy for AB disease management. Although many QTLs have been successfully used to improve AB resistance, genes controlling resistance mechanisms are still unknown. More studies are needed to narrow down regions associated with AB resistance in chickpea and facilitate the development of marker-assisted selection (MAS) strategies. These techniques can be used by breeders to pyramid multiple QTLs to improve AB resistance in chickpea. This study aimed to: (1) construct a genetic map and identify QTLs associated with AB resistance in two recombinant inbred line (RIL) populations of chickpea against different AB races from Lebanon and India and (2) identify candidate genes for AB resistance.

## Materials and Methods

### Plant Materials

Two chickpea intraspecific RIL populations (AB_3279_ and AB_482_) were used to identify genomic regions associated with resistance to AB. AB_3279_ contains 116 RILs (F_10_) derived from a cross between “ILC1929” and “ILC3279.” AB_482_ contains 135 RILs (F_10_) derived from a cross between “ILC1929” and “ILC482.” “ILC1929” is a Kabuli landrace collected by ICARDA that originated from Syria and is susceptible to AB. “ILC3279” is a Kabuli landrace collected by ICARDA that originated from the former Union of Soviet Socialist Republics (USSR) and is resistant to AB pathotypes I and II (Udupa et al., 1998; Udupa and Baum, 2003). “ILC482” is a Kabuli landrace collected by ICARDA that originated from Turkey and has a moderate level of resistance to AB pathotype I (Udupa et al., 1998).

### Phenotyping for *Ascochyta* Blight Resistance

The two RIL populations, AB_3279_ and AB_482_, and the three parents, “ILC929,” “ILC3279,” and “ILC482,” were screened for their response to AB under controlled greenhouse and field conditions in Lebanon and India during the cropping seasons of 2015, 2017, and 2018. In all locations, there are commonly used chickpea varieties included as checks for *Ascochyta* screening susceptible (ILC263 and Sel74102 genotypes).

#### Multiplication of Pathogen

In Lebanon, the inocula of two *A. rabiei* isolates, AR-01 (pathotype-1, mildly aggressive) and AR-02 (pathotype-2, moderately aggressive), were prepared in the Pulse Pathology Laboratory of ICARDA Terbol. These two isolates are routinely used to screen ICARDA chickpea breeding lines for AB resistance under field and controlled conditions (Udupa et al., 1998; Imtiaz et al., 2011). The plants in the greenhouse and field experiments were artificially inoculated by spraying pathotypes I and II (1998). In India, isolate 8, race 3968 (designated by Singh, 1990) was prepared at Punjab Agricultural University (PAU), Ludhiana, for artificial inoculation of the field nursery. The isolate was maintained on potato dextrose agar (PDA, 200 g potato, 20 g dextrose, 20 g agar, and 1 L H_2_O) slants at Pulses Pathology Laboratory, Department of Plant Breeding and Genetics and multiplied on potato dextrose broth (200 g potato, 20 g dextrose, and 1 L H_2_O) at 22°C for use in artificial field inoculations.

#### Greenhouse Screening

In the greenhouse at the ICARDA station, Terbol, Lebanon, five surface-sterilized chickpea seeds were planted in plastic pots (12 cm in diameter) filled with sterilized soil. The temperature was adjusted to 18–20°C, with a photoperiod of 16 h artificial light. The five plants were evaluated in two replications arranged in a completely randomized block design. The entire experiment was repeated twice against pathotypes I (T-SR-I) and II (T-SR-II). The inoculum was prepared from 7-day old cultures, and spore suspension was adjusted to 5 × 10^5^ ml^–1^ using a cell counter. About 2-week-old seedlings in the 4- to 6-leaf stage were inoculated with a spore suspension of *Ascochyta rabiei* isolates of pathotypes I and II separately, and each pot was sprayed until runoff using a motorized sprayer.

#### Field Screening

The five plants were screened for AB responses under field conditions in the test locations of Kfarshakhna (KSH) station (in the Lebanese coastal area) (latitude: 34.36, longitude: 35.86, altitude: 203 m) in years 2015 and 2018 (KSH-15 and KSH-18) and at the ICARDA station, Terbol, Lebanon (latitude: 33.81, longitude: 35.99, altitude: 894 m). In India, in 2017, artificial epiphytotic field conditions were created by artificial inoculations and using sprinkler systems at PAU in Ludhiana. Each RIL population, during the field screening, was arranged in an unbalanced alpha lattice design with two replications in all locations (Supplementary Material 1).

#### Observations

Response to AB disease in the greenhouse screening and field screening experiments in Lebanon and India was evaluated 2 and 4 weeks after inoculation using the 1- to 9-point rating scale (1 = no symptoms, 9 = susceptible) developed by Gurha et al. (2003; Table 1).

**TABLE 1:** Disease rating scale for *Ascochyta* blight (AB) in chickpea.

Scale	Disease intensity	Reaction
1	No disease visible on any plant	Highly resistant
3	Lesions visible on <10% of the plants, no stem girdling	Resistant
5	Lesions visible on up to 25% plants, stem girdling on <10% plants but little damage	Moderately resistant
7	Lesions present on most of the plants, stem girdling on 50% of the plants and resulting death of a few plants causing considerable damage	Susceptible
9	Lesions profuse on all plants stem girdling present on >50% of plants and death of most of the plants	Highly susceptible

*The AB_3279_ and AB_482_ populations were scored for disease reaction on a 1–9 rating scale. Lines with a disease rating of ≤4 were considered resistant, and lines with disease rating of 5–6 were considered moderately resistant; and those with rating of above 6 were considered susceptible to highly susceptible.*

### Statistical Analyses

Statistical analyses were conducted using the GenStat software (version 19.1; VSN International Ltd., Hemel Hempstead, United Kingdom). In the greenhouse experiment, individual analyses for the two greenhouse experiments against pathotypes I and II were conducted. ANOVA was performed in which genotypes were considered as a fixed factor and replications were considered as a random factor to compute the average AB susceptibility score for each RIL against each pathotype. For the field experiment, analyses were conducted in two ways: (1) combined analyses across years and locations, and (2) separate analyses for each available combination of the years and locations Kfarshakhna-2015 (K-SH-2015), Punjab Agricultural University-2017 (PAU-2017), and Kfarshakhna-2018 (KSH-2018) were conducted for each population separately (AB_3279_ and AB_428_). Environmental conditions were suboptimal for disease development at the Terbol location in season 2015; hence, the data collected that season were excluded from analysis. The residual maximum likelihood (REML) method was used with VCOMPONENTS in which genotypes were considered as a fixed factor and environment and replication as random factors (environment is a combination of year and location). VPREDICT was used to compute the average AB susceptibility score for each RIL according to Singh and Ceccarelli (1995). Pearson’s correlation coefficients were calculated between field and greenhouse disease data.

### Generation of Single Nucleotide Polymorphism Marker by Genotyping-by Sequencing

DNA extraction and library construction and sequencing were conducted at the Centre of Excellence in Genomics (CEG), International Crops Research Institute for the Semi-Arid Tropics (ICRISAT), Patancheru, India. DNA isolation was carried out for RILs and the parental genotypes for both populations, using the high-throughput mini-DNA extraction method and the NucleoSpin^®^ 96 Plant II Kit (MACHEREY-NAGEL Company). The quality of DNA was assessed using a spectrophotometer (Shimadzu UV160A, Japan). Samples were subjected to the GBS approach for SNP identification as described by Elshire et al. (2011). GBS libraries from parental lines and RILs without adapter dimers were sequenced using the Illumina HiSeq 2500 platform (Illumina Inc., San Diego, CA, United States).

#### Single Nucleotide Polymorphism Calling

Processing of NGS reads and SNP calling procedure were conducted using TASSEL-GBS pipeline version 4.0 according to pipeline documentation (Bradbury et al., 2007; Glaubitz et al., 2014). Reads having more than 50% of low-quality base pairs (Phred < 5%) were discarded, and filtered data were used for calling SNPs after a quality check (Q score > 20). Master tags (i.e., collapsed sequence tags from each sequence file) were aligned along the draft genome sequence (CaGAv1.0) of chickpea (Varshney et al., 2013). The nucleotide with highest probability in each position under a Bayesian model was identified for individual RILs, and consensus sequences were saved in FASTA format. Consensus sequences from all the samples were compared to detect polymorphic loci. Polymorphic loci that were either heterozygous in any of the parents or present in <50% of individuals in the population were discarded, and a high-quality SNP data set was generated.

#### Linkage Mapping

Genotyping data for SNPs generated with the GBS approach were compiled for linkage analysis using JoinMap V4.0 (Van Ooijen and Voorrips, 2006). Marker order was assigned using a regression mapping algorithm with a maximum recombination frequency of 0.4 at a minimum logarithm of odds (LODs) of 3 and a jump threshold of 5. The Ripple command was used after adding each marker locus to confirm marker order. The Kosambi mapping function was used to calculate map distance (Kosambi, 1943). To detect segregation distortion, chi-square (χ^2^) values were calculated using Joinmap V4.0. Highly distorted and unlinked markers were excluded from the analysis. Mapchart 2.2 (Voorrips, 2002) was used to visualize the constructed map for each linkage group. Linkage groups were named according to Varshney et al. (2014a).

#### Quantitative Trait Loci Analysis and Gene Annotation

The genotyping data obtained from this study and the phenotyping data for both populations for AB resistance were used for QTL analysis using the QTL Cartographer V.2.5 software (Wang et al., 2012). Composite interval mapping (CIM) was performed by selecting model 6 with default window size 10 cM, control marker number 5, and the backward regression method. To obtain more precise results, default walk speed was reduced to 1 cM. The LOD method (LOD > 3) was used to determine the significance of each QTL interval with the threshold level performed at 1,000 permutations, and significance level was *p* ≤ 0.05. QTLs were considered “stable” (if they appeared in more than one location for the specified trait) or “consistent” (if they appear in more than 1 year/season for the specified trait) as described in Varshney et al. (2014b). The Circos package (Krzywinski et al., 2009) was used to plot the concentration of SNP markers on the chickpea genome using in-home PERL scripts. Genes located in the QTL region delimited through the GBS approach were retrieved from the draft genome sequence (CaGAv1.0) of chickpea (Varshney et al., 2013). Gene enrichment analysis (GEA) was conducted using the PANTHER database (Thomas et al., 2003), and the protein-protein interaction (PPI) network was assessed using the STRING database (Mering et al., 2003).

## Results

### Resistance to *Ascochyta* Blight in the Two Populations AB_3279_ and AB_482_

Environmental conditions played an important role in disease development and progression in the field experiments (Supplementary Figure 1). The variance component analysis, under field conditions, showed significant differences in AB severity for both RILs populations (AB_3279_ and AB_482_) in both combined and individual year and location analyses (*p* < 0.001; Table 2). The data for the second population under greenhouse conditions against pathotype II and at Kfarshakhna in 2018 were suboptimal and, hence, were excluded from the analysis of variance or regression and QTL analysis.

**TABLE 2:** Analysis of variance for AB scores of the chickpea recombinant inbred line (RIL) AB_3279_ and AB_482_ populations under greenhouse conditions (against pathotypes I and II) and under field conditions at Kfarshakhna (KSH) in 2015 and 2018 and Punjab Agricultural University (PAU) in 2016.

Year/locations/environments		AB_3279_	AB_482_
Greenhouse against P I	Grand mean	4.720	4.66
	G (*P* value)	<0.001	<0.001
	SE	0.89	0.78
	LSD	1.76	2.18
	CV%	18.9	23.7
	H^2^	0.87	0.84
Greenhouse against P II		AB_3279_	AB_482_
	Grand mean	7.33	
	G (*P* value)	<0.001	
	SE	0.58	
	LSD	1.64	
	CV%	11.3	
	H2	0.78	
Field combined environments		AB_3279_	AB_482_
(KSH-2015, PAU-2016 and KSH-2018 only for population AB3279)	E (*P* value)	<0.001	<0.001
	G (*P* value)	<0.001	<0.001
	GE (*P* value)	0.59	0.20
	E (Av. SE)	000	0.28
	G (Av. SE)	0.73	0.96
	GE (Av. SE)	1.27	1.37
Individual year/location		AB_3279_	AB_482_
KSH-2015	Grand mean	6.90	8.23
	G (*P* value)	<0.001	<0.001
	Av. SE	0.62	0.47
	Av. LSD	1.74	1.32
	CV%	12.8	8.1
	H2	0.81	0.78
PAU-2017	Grand mean	5.56	5.65
	G (*P* value)	<0.001	0.002
	Av. SE	1.26	1.26
	Av. LSD	3.54	3.51
	CV%	32.2	31.5
	H2	0.62	0.58
KSH-2018	Grand mean	7.11	
	G (*P* value)	<0.001	
	Av. SE	0.66	
	Av. LSD	1.87	
	CV%	13.3	
	H2	0.80	

*G, genotypes of individual RILs from the AB_3279_ and AB_482_ populations; E, environments; GE, genotype by environment interaction. Av. SE is average standard error; Av. LSD is average least significant differences; CV% is coefficients of variation; and H^2^ is broad-sense heritability. K-SH, PAU.*

**FIGURE 1:**
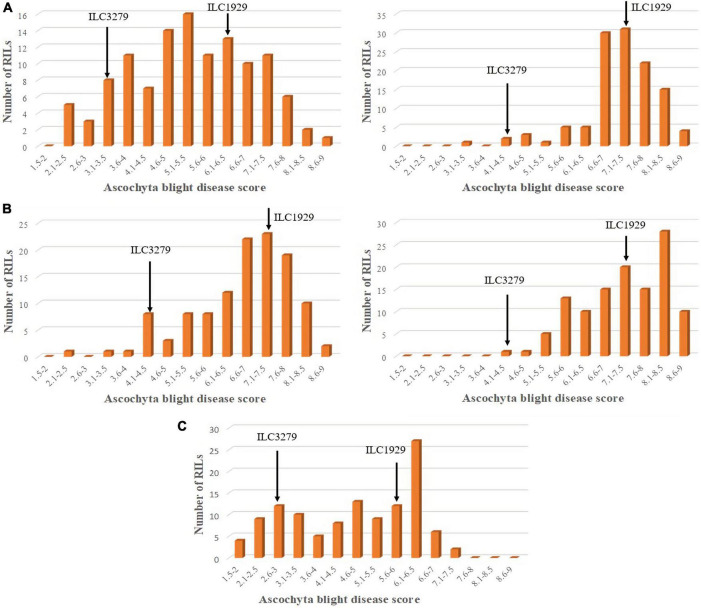
Frequency distribution of *Ascochyta* blight (AB) disease scores in 116 recombinant inbred lines (RILs) of the AB_3279_ chickpea population developed from a cross between “ILC1929” and “ILC3279.” The mean disease score was used to calculate the frequency distribution of disease severity for pathotypes I and II under greenhouse **(A)**, and fields conditions at Kfarshakhna in 2015 and 2018 **(B)**, and Punjab Agricultural University (PAU) Ludhiana in 2017 **(C)**. The arrows represent the mean scores of the resistant “ILC3279” and susceptible “ILC 1929” parents.

In the greenhouse experiments for the first population AB_3279_, AB scores ranged from 6.2 to 7.5 for the susceptible parent “ILC1929,” and from 3.1 to 4.5 for the resistant parent “ILC3279” against pathotypes I and II, respectively. Average AB scores of the AB_3279_ population were 4.7 and 7.4 and ranged from 1.2 to 7.2 and from 3.1 to 9, respectively, for pathotypes I and II (Figure 1). The coefficients of variation (CV) of AB disease score under greenhouse conditions were 11.3 and 18.9% against pathotypes I and II, respectively (Table 2). Field disease screenings of this RIL population were conducted in the flowering stage at KSH in 2015 and 2018 and at PAU in 2017, and significant differences were detected among the RILs (Figure 1). The average AB scores for the resistant parent “ILC3279” were 4.5, 4.1, and 2.7, whereas the average scores of the susceptible parent “ILC1929” were 7.5, 7.5, and 5.5 at KSH in 2015 and 2018, and at PAU in 2017, respectively.

**FIGURE 2:**
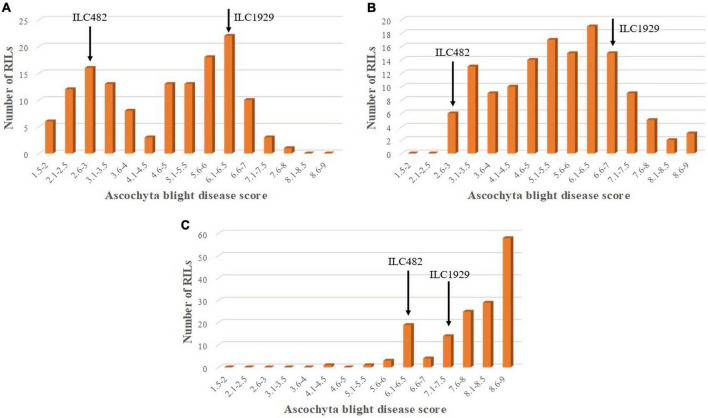
Frequency distribution of AB disease scores in 135 RILs of the AB_482_ chickpea population developed from a cross between “ILC1929” and “ILC482.” Mean disease score was used to calculate the frequency distribution of disease scores in the greenhouse experiment repeats for pathotype I under **(A)** field conditions at Kfarshakhna in 2015 and **(B)** at PAU Ludhiana in 2017. **(C)** Arrows show the mean scores of the resistant “ILC482” and susceptible “ILC929” parents.

The AB scores of the parents of the RIL population AB_482_ under greenhouse conditions against pathotype I were 2.6 for the resistant parent “ILC482” and 6.2 for the susceptible parent “ILC1929,” respectively. Overall, in the population AB_482_, significant differences were detected among the RILs, with an average AB score of 4.7, a range of 1.6–7.5 (Figure 2), and a coefficient of variation of 23.7% (Table 2). For the field screening of this RIL population, the average AB scores for the resistant parent ILC482 were 6.4 at KSH in 2015 and 2.5 at PAU in 2017, while the respective scores for the susceptible parent “ILC1929” were 7.3 and 6.9.

For pathotype II in the greenhouse screening, the AB scores of the resistant parent “ILC482” and the susceptible parent “ILC1929” were 8.5 and 9, respectively. Under field conditions at KSH in the 2018 season, disease development was higher than at KSH during the 2015 season, because the conditions were more conducive to the development of pathotype II than pathotype I (Figure 2). At KSH 2015, the AB scores of the resistant parent “ILC 482” and the susceptible parent “ILC1929” were 7 and 7.5, respectively. The distribution of disease scores for all the locations and years did not follow a normal distribution, and the disease scores extended beyond those of the parents in both populations AB_3279_ and AB_482_, suggesting transgressive segregation (Figures 1, 2).

The interaction of genotype by environment (location and year) was significant; therefore, data from the field experiments were not combined, and the final disease scores from individual locations and years were used separately for QTL analyses.

Broad-sense heritability (H^2^) estimates were very high for both populations under greenhouse and field conditions. The heritability for the AB_3279_ population was high, with values of 0.87 and 0.78 for pathotypes I and II under greenhouse conditions, respectively, and were 0.81, 0.80, and 0.62 under field screenings at KSH-2015, KSH-2018, and PAU-2017, respectively. For the AB_482_ population, the heritability was 0.84 under greenhouse conditions against pathotype I, and 0.78 and 0.58 under field screenings at KSH-2015 and PAU-2017, respectively (Table 2).

### Single Nucleotide Polymorphism Genotyping and Linkage Mapping Analysis

**FIGURE 3:**
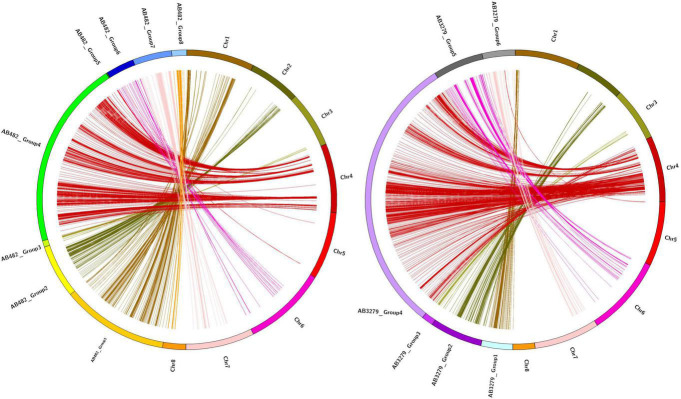
Chromosomal distribution of single nucleotide polymorphism (SNP) markers in the chickpea genome, where **(A)** is the density of the AB_482_ population (per 1 Mbp), **(B)** is the density of the AB_3279_ population (per 1 Mbp), and (D) is the marker logarithm of odds (LOD) score (red points) only for AB_3279_.

Approximately 600 million raw reads with an average of approximately 230 MB per sample were generated in the two RIL populations, AB_3279_ and AB_482_. In total, 2,736 (4.19%) polymorphic markers were identified between the two parents of the AB_3279_ population, of which 2,074 were mapped on eight linkage groups (CaLG01–CaLG08) covering 3,736.63 cM with an average marker density of 1.8 cM. Uneven distribution of SNP markers in chickpea linkage groups was observed, with CaLG04 having the highest number of SNPs (1,265) while CaLG05 had the lowest number of SNPs (only 29). The average length of the 8 linkage groups was 467.08 cM, (Table 3 and Figure 3). The AB_482_ population had 2,080 (4.13%) polymorphic SNPs between the two parents; of these, 1,652 SNPs were mapped on the eight linkage groups (CaLG01–CaLG08) covering 3,242.16 cM with an average marker density of 1.96 cM. Uneven distribution of SNP markers in the chickpea linkage groups was observed, with CaLG04 having the highest number of loci (814 SNPs) while CaLG05 had the lowest (only 16 SNPs). The average length of the eight linkage groups was 405.27 cM (Table 3 and Figure 3). The low number of detected SNPs in CaLG5 could be due to the quality control step, which allowed us to consider only QTLs with high quality and high calling rates. Additionally, the linkage mapping discarded unlinked markers.

**TABLE 3:** Distribution of markers on the intra-specific genetic map based on both chickpea RIL populations AB_3279_ and AB_482_.

Linkage groups	Number of SNP markers	Map distance ^a^(cM)	Average distance between two markers
AB_3279_			
CalG01	136	235.56	1.7
CalG02	176	390.21	2.2
CalG03	61	86.65	1.4
CalG04	1,265	2,102.32	1.7
CalG05	29	188.90	6.5
CalG06	193	374.53	1.9
CalG07	87	243.88	2.8
CalG08	127	114.57	0.9
Total	2,074	3,736.63	1.8
AB_482_			
CalG01	321	761.62	2.4
CalG02	201	380.92	1.9
CalG03	55	43.59	0.8
CalG04	814	1,347.90	1.7
CalG05	16	124.60	7.8
CalG06	41	201.16	4.9
CalG07	130	277.35	2.1
CalG08	74	105.02	1.4
Total	1,652	3,242.16	1.96

*^a^cM, centiMorgan.*

### Identification of Quantitative Trait Loci Associated With *Ascochyta* Blight Resistance

#### Quantitative Trait Loci for *Ascochyta* Blight Resistance in the AB_3279_ Recombinant Inbred Population

A total of 393 SNPs located in QTL regions for AB resistance explaining 11.2–39.3% of the phenotypic variation (PVE) were identified on CalG02 and CalG04. Notably, all alleles in the AB resistance loci showed negative additive effects originating from the parent “ILC1929,” which indicates that the resistance in the other parent “ILC3279” is recessive, and that the increase in resistance is due to the absence of alleles in this parent. A QTL analysis based on 176 markers mapped on CalG02 and phenotypic data for AB resistance identified thirty-two AB-associated SNPs with LOD scores ranging from 3 to 6.17 (Supplementary Figure 2).

A QTL analysis based on 1,265 SNPs mapped on CalG04 identified 361 AB-associated SNPs located in QTLs regions (including all SNPs of LOD ≥ 3), which individually explained 11.2–39.3% of the phenotypic variation (PVE), with LOD as high as 12.57 pertaining to AB resistance (Supplementary Figure 3).

Nine QTLs were detected in CalG02 and CalG04, and two QTLs were detected in CalG02 (Table 4). The QTL named AB_3279_-LG2-qtl-1, pertaining to AB resistance under field conditions at the KSH-2015 and KSH-2018 locations and under controlled environment against pathotype, was located at 237.53–255.77 cM (18.24 cM, 3.3 kb), corresponding to the physical region at SNP-30915967 to SNP-30912678 bp. The second QTL named AB_3279_-LG2-qtl-2 spanned from 220.86 to 234.63 cM (13.77 cM, 373 kb), corresponding to the physical region at SNP-32481969 to SNP-32108629 bp.

**TABLE 4:** Quantitative trait loci (QTLs) associated with AB resistance identified in ILC3279 × ILC1929 chickpea population in chromosome CalG02.

QTLs	Environment	Interval (cM^a^–bp^b^)	Flanking marker
AB_3279_-LG2-QTL-1	TSR-PI	220.86–234.63 (13.77–373 Kb^c^)	32481969–32108629
AB3279-LG2-QTL-2	KSH-15, KSH-18, T-SR-PI	237.53–255.77 (18.24–3.2 Kb)	30915967–30912678

*KSH-15, Kfarshakhna 2015; KSH-18, Kfarshakhna 2018; TSR-PI, seedling resistance in Terbol Pathotype I.*

*^a^cM, centimorgan.*

*^b^bp, basepair.*

*^c^KB, kilobasepair.*

Four stable and consistent QTLs were detected in CalG04 (Table 5). Details of some of these QTLs are as follows: AB_3279_-LG4-qtl-1 was identified across five different environments under field (KSH-15, KSH-18, and PAU) and controlled conditions (T-SR-PI and T-SR-PII). AB_3279_-LG4-cluster-1 was located at 43.08–56.3 cM (13.2 cM, 4.8 kb), corresponding to the physical region at SNP-4913945 to SNP-4909163 bp.

**TABLE 5:** Quantitative trait loci associated with AB resistance identified in ILC3279 × ILC1929 population on chromosome CalG04.

QTLs	Environment	Interval (cM^a^–bp^b^)	Flanking marker
AB**_3279_**-LG4-QTL-1	KSH-15, KSH-18, PAU, SR-PI, SR-PII	43.08–56.3 (13.22–4.7 Kb^c^)	4913945–4909163
AB**_3279_**-LG4-QTL-2	KSH-15, KSH-18, PAU, SR-PII	60.53–79.07 (18.5–151 Kb)	4142264–3991658
AB**_3279_**-LG4-QTL-3	KSH-18	160.67–175.68 (15–175 Kb)	8677597–8852420
AB**_3279_**-LG4-QTL-4	SR-PI	895.48–918.35 (22.87–265 Kb)	36977280–36712806
AB**_3279_**-LG4-QTL-5	KSH-18, PAU, SR-PI	928.26–942.95 (14.69–1.2 Mb)	37700732–38921338
AB**_3279_**-LG4-QTL-6	PAU	1597.96–1626.03 (28–20 Kb)	15962223–15942274
AB**_3279_**-LG4-QTL-7	KSH-18, PAU, SR-PI	1998.66–2019.45 (20.8–299 Kb)	10974975–11274281

*PAU, Punjab Agricultural University; KSH-2015, Kfarshakhna 2015; KSH-2018, Kfarshakhna 2018; SR-PI, seedling resistance in Terbol pathotype I; SR-PII, seedling resistance in Terbol pathotype II.*

*^a^cM, centimorgan.*

*^b^bp, base pair.*

*^c^KB, kilobase pair.*

CalG04 harbored three QTLs in different environments (Table 5). AB_3279_-LG4-QTL-3, identified in KHS-18, spanned from 160.67 to 175.68 cM (15 cM, 175 kb), corresponding to the physical region at SNP-8677597 to SNP-8852420 bp. Details for the other QTLs are given in Table 5.

#### Quantitative Trait Loci for *Ascochyta* Blight Resistance in AB_482_ (ILC482 × ILC1929) Recombinant Inbred Population

A QTL analysis based on 1,649 SNPs mapped on CalG04 and phenotypic data for AB resistance identified a total of 256 AB-associated SNPs located in QTLs regions (including all SNPs of LOD ≥ 3), which individually explained 9.7–35% of the phenotypic variation (PVE), with LOD as high as LOD = 12.64 for AB resistance (Supplementary Figure 4). Notably, all alleles in the AB-resistant loci showed positive additive effects originating from “ILC482,” which indicates that the increase in resistance is due to the presence of the alleles from “ILC482,” and that the resistance was dominant.

In this population, nine QTLs were detected in CalG04 (Table 6). Details of some of these QTLs are as follow: AB_482_-LG4-QTL-7 was identified under field conditions (PAU and KSH-15) and the controlled environment against pathotype I (SR-PI). AB_482_-LG4-QTL-7 was located at 1,141.14–1,170 cM (28.9 cM, 48 kb), corresponding to the physical region at SNP-4737543 to SNP-4689032 bp; AB_482_-LG4-QTL-1 was identified under controlled environment against pathotype I (SR-PI) and was located at 280.89–301.42 cM (20.53 cM, 286 kb), corresponding to the physical region at SNP-38609927 to SNP-38324265 bp. Details for the QTLs are given in Table 6.

**TABLE 6:** Quantitative trait loci associated with AB resistance identified in the ILC482 × ILC1929 population of chickpea in chromosome CalG04.

QTLs	Environment	Interval (cM^a^–bp^b^)	Flanking marker
AB_482_-LG4-QTL-1	SR-PI	280.89–301.42 (20.53–287 Kb^c^)	38609927–38324265
AB_482_-LG4-QTL-2	SR-PI	363.05–389.02 (25.97–730 Kb)	38079511–37349331
AB_482_-LG4-QTL-3	SR-PI	617.6–644.38 (26.78–695 Kb)	16822181–16127324
AB_482_-LG4-QTL-4	SR-PI	707.93–736.73 (28.8–191 Kb)	15934568–15743709
AB_482_-LG4-QTL-5	PAU, SR-PI	794.9–806.84 (11.94–283 Kb)	11491576–11774322
AB_482_-LG4-QTL-6	PAU, SR-PI	926.07–945.6 (19.53–146 Kb)	10931884–11077419
AB_482_-LG4-QTL-7	PAU, SR-PI, KSH-15	1141.14–1170 (28.9–48 Kb)	4737543–4689032
AB_482_-LG4-QTL-8	KSH-15, PAU	1201.17–1228 (26.9–174 Kb)	4320745–4254747
AB_482_-LG4-QTL-9	PAU, SR-PI, KSH-15	1230.86–1259.44 (29–7 Kb)	4244918–4251864

*PAU, Punjab Agricultural University; KSH-15, Kfarshakhna-2015; KSH-18, Kfarshakhna-2018; SR-PI, seedling resistance in Terbol pathotype I.*

*^a^cM, centimorgan.*

*^b^bp, base pair.*

*^c^KB, kilobase pair.*

### Physical Mapping of Genomic Regions Associated With Resistance to *Ascochyta* Blight

Sequences of the regions flanking the SNPs were used to anchor the QTLs to the chickpea physical map. The physical mapping of SNPs markers linked to AB resistance has led to the identification of four genomic regions in CalG02, 13 genomic regions in CalG04 in the AB_3279_ population, and 11 genomic regions in CalG04 in the AB_482_ population.

#### Common Genomic Regions Associated With *Ascochyta* Blight Resistance in “ILC3279” and “ILC482”

**FIGURE 4:**
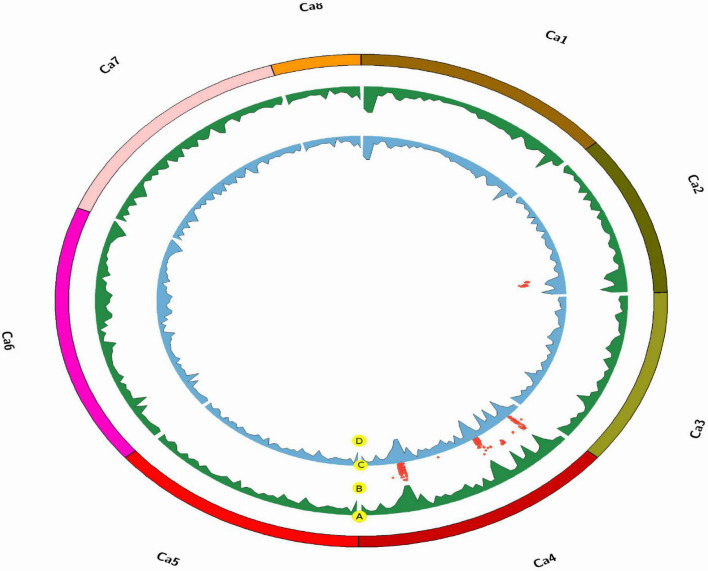
Chromosomal distribution of SNP markers in the chickpea genome, where **(A)** is the density of the AB_482_ population (per 1 Mbp), **(B)** is the marker LOD score (red points) between both populations, **(C)** is the density of the AB_3279_ population (per 1 Mbp), **(D)** is the marker LOD score (red points) only for AB_3279_.

**FIGURE 5:**
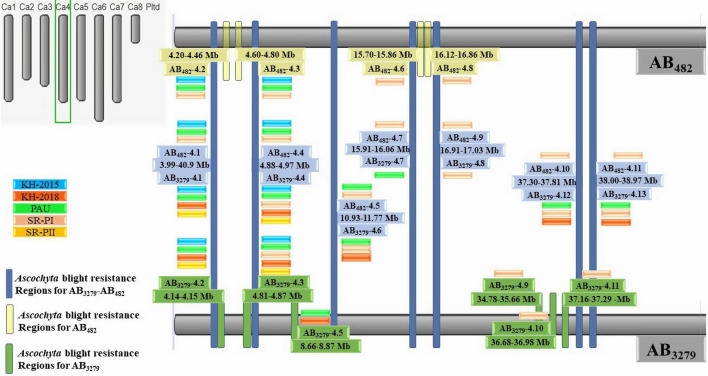
High-density intraspecific genetic map of chickpea (ILC482 × ILC1929) using genotyping-by-sequencing (GBS)-SNP markers. Bars in dark blue color font are the common genomic regions in both populations AB_3279_ and AB_482_, bars in yellow color font are genomic regions in AB_482_. Bars in green color font are genomic regions in AB_3279_.

“ILC3279” and “ILC482” are two moderately resistant cultivars having different genetic backgrounds. However, QTLs on chromosome CaLG04 were identified in common genomic regions of both populations. Seven major genomic regions were common between the AB_3279_ and AB_482_ populations in different environments (Figures 4, 5). The genomic region AB_3279–482_.1 (CaLG04: 3990334–4098404 ∼108 kb) containsAB_3279_-4.1 and AB_482_-4.1, significantly associated with AB resistance in seven different environments for both populations AB_3279_ (PAU, KSH-2015, KSH-2018, and SR-PII) and AB_482_ (PAU, KSH-2015, and SR-PI).

Another genomic region, AB_3279–482_.2 (CaLG04: 4882803–4974830 ∼92 kb), contains AB_3279_-4.4 and AB_482_-4.4, significantly associated with AB resistance in different environments for both populations AB_3279_ (PAU, KSH-2015, KSH-2018, SR-PI, and SR-PII) and AB_482_ (PAU, KSH-2015, and SR-PI). The genomic region AB_3279–482_.3 (CaLG04: 10931884–11771922 ∼840 kb) contains AB_3279_-4.6 and AB_482_-4.5, significantly associated with AB resistance in five different environments for both populations AB_3279_ (PAU, KSH-2018, and SR-PI) and AB_482_ (PAU and SR-PI).

The region AB_3279–482_.4 (CaLG04: 15933422–16076690 ∼143 kb) contains AB_3279_-4.7 and AB_482_-4.7 detected in two different environments (SR-PI) and AB_482_ (PAU). One region, AB_3279–482_.5 (CaLG4: 16990705–17037384 ∼47 kb) containing AB_3279_-4.8 and AB_482_-4.9, was detected in only a controlled environment against pathotype I (SR-PI). Two regions in CaLG4 were detected in four different environments in both populations AB_3279_ (PAU, KSH-2018, and SR-PI) and AB_482_ (SR-PI); the first region, AB_3279–482_.6 (CaLG04: 38008706–38979508 ∼970 kb) contained AB_3279_-4.13 and AB_482_-4.11 and the second region, AB_3279–482_.7 (CaLG04: 37348105–37818047 ∼470 kb), contained AB_3279_-4.12 and AB_482_-4.10.

#### Specific Genomic Regions Associated With Resistance to *Ascochyta* Blight in “ILC3279”

Four major genomic regions in CalG02 regulating AB resistance in the genotype “ILC3279” in different environments were identified (Figures 4, 5). The first region was detected in three different environments (KSH-2015, KSH-2018, and SR-PI) and called AB_3279_-2.1, which spanned CaLG02: 30902858–30997784 ∼95 kb and contained 24 SNPs explaining 11.2–15.7% of the phenotypic variation. Two regions, AB_3279_-2.2 and AB_3279_-2.4, played a role in the resistance shown in two different environments (KSH-2018 and SR-PI); AB_3279_-2.2 contained 32 SNPs significantly associated with AB resistance explaining 12.2–21.7% of the phenotypic variation and spanned CaLG02: 32108597–32108629 ∼32 pb; AB_3279_-2.4 spanned over CaLG02: 32432904–32488272 ∼55 kb and contained 10 SNPs significantly associated with AB resistance explaining 11.5–19.7% of the phenotypic variation. Only one region was identified under controlled conditions against pathotype I, called AB_3279_-2.3, which spanned over CaLG02: 32109805–32415818 ∼306 kb and contained 37 SNPs significantly associated with AB resistance and explaining 13.5–18.1% of the phenotypic variation.

Thirteen genomic regions were identified in CalG04 in the population AB_3279_; seven of them were common with the population AB_482_, and six were specific to the population AB_3279_. The region AB_3279_-4.2 was detected in different environments (PAU, KSH-2015, KSH-2018, and SR-PII), but no SNPs pertaining to AB resistance in controlled environment against pathotype I were detected in the region AB_3279_-4.2 (CaLG04: 4141107–4153455 ∼12.3 kb). This region contained 17 significantly associated SNPs with AB resistance explaining 11.2–24.1% of the phenotypic variation.

The other region with a role in the resistance, AB_3279_-4.3, spanned on physical map CaLG04: 4811896–4872102 ∼60 kb and was detected in different environments (PAU, KSH-2015, KSH-2018, SR-PI, and SR-PII), and this region included 31 SNPs explaining 11.3–33.7% of the phenotypic variation.

The region AB_3279_-4.5 was identified under field conditions in two different locations in KSH-2018 and PAU. This region spanned CaLG04: 8660115–8875933 ∼216 kb and included 35 SNPs that were significantly associated with AB resistance and explaining 11.3–15% of the phenotypic variation.

The physical mapping of SNP markers led to the identification of three major genomic regions controlling resistance to AB in the cultivar “ILC3279” in a controlled environment against pathotype I (SR-PI) only. The first one, AB_3279_-4.9 (CaLG04: 34784962–35664917 ∼880 kb) contained 56 SNPs explaining 11.3–15.1% of the phenotypic variation. The second region, AB_3279_-4.10 (CaLG04: 36368625–36983721 ∼615 kb), contained 58 SNPs explaining 11.5–19.7% of the phenotypic variation. The third region, AB_3279_-4.11 (CaLG04: 37160576–37293408 ∼133 kb) contained 26 SNPs explaining 11.3–22.2% of the phenotypic variation.

#### Specific Genomic Regions Associated With Resistance to *Ascochyta* Blight in “ILC482”

Eleven genomic regions controlling AB resistance were identified in the population AB_482_, and all were located in CalG04 (Figures 4, 5). Seven of them were common with regions in the population AB_3279_, and four regions were specific to the population AB_482_.

Two genomic regions were detected under field environments (PAU and KSH-2015) and controlled environment pathotype I (SR-PI): The first region called AB_482_-4.2 spanned on physical map CaLG04: 4205361–4468395 ∼263 kb and included 100 SNPs explaining 9.7–28.6% of the phenotypic variation; the second region spanned CaLG04: 4605334–4805677 ∼200 kb, and was called AB_482_-4.3; it included 79 SNPs significantly associated with AB resistance and explaining 9.8–29.7% of the phenotypic variation.

Two major regions with high phenotypic variation were detected in the parent “IL482” under controlled conditions against pathotype I (SR-PI), and only AB_482_-4.6 spanned CaLG04: 15701299–15860311 ∼159 kb, with 37 SNPs significantly associated with AB resistance that explained 23.9–37% of phenotypic variation; AB_482_-4.8 spanned CaLG04: 16127324–16865990 ∼739 kb with 55 SNPs significantly associated with AB resistance and explaining 20.3–30.4% of the phenotypic variation.

#### New Genomic Regions Associated With Resistance to *Ascochyta* Blight in “ILC3279” and “ILC482”

In recent years, several QTLs and genomic regions have been reported to be associated with AB resistance. In our study, we have identified 21 genomic regions associated with AB resistance on CaLG02 and CaLG04 in chickpea. Among the identified regions, 12 genomic regions were shared with previous AB studies, while nine were unique to our study (Supplementary Table 1). The shared genomic regions were located in CaLG02 and CaLG04, while the unique genomic regions were located only in CaLG04. The nine distinct regions linked with our study contain 819 SNPs that are significantly associated with AB resistance in different environments, and of which five were shared by two populations, three were specific to AB_3279_, and one was specific to AB_482_. The proposed physical map narrowed down regions identified in previous studies as conferring AB resistance consistent genomic regions across different environments.

### Pathotype-Specific Regions for *Ascochyta* Blight Resistance

The two populations, AB_3279_ and AB_482_, showed significantly different resistance patterns to *A. rabiei* pathotypes I and II in a controlled environment and race populations under field conditions. All eleven regions (AB_482_-4.1, AB_482_-4.2, AB_482_-4.3, AB_482_-4.4, AB_482_-4.5, AB_482_-4.6, AB_482_-4.7, AB_482_-4.8, AB_482_-4.9, AB_482_-4.10, and AB_482_-4.11) were detected in the genotype “ILC482” in the controlled environment against pathotype I, out of which four regions (AB_482_-4.1, AB_482_-4.2, AB_482_-4.3, and AB_482_-4.4) were identified under field conditions in India (PAU) and in Lebanon at KSH-2015 using a mixture of pathotypes I and II, and one region was detected under field conditions at PAU.

Four genomic regions (AB_3279_-2.1, AB_3279_-2.2, AB_3279_-2.3, and AB_3279_-2.4) were identified in CaLG02-resistant AB in a controlled environment against pathotype I in the genotype “ILC3279,” and one of them (AB_3279_-2.3) was specific for pathotype I, and another (AB_3279_-2.1) was detected under field conditions in Lebanon at KSH-2015 and KSH-018. The remaining two regions (AB_3279_-2.2 and AB_3279_-2.4) were identified under field conditions in Lebanon at KSH-2018.

Nine genomic regions (AB_3279_-4.3, AB_3279_-4.4, AB_3279_-4.6, AB_3279_-4.8, AB_3279_-4.9, AB_3279_-4.10, AB_3279_-4.11, AB_3279_-4.12, and AB_3279_-4.13) were detected in the genotype “ILC3279” in a controlled environment against pathotype I; four of which were specific to pathotype I (AB_3279_-4.8, AB_3279_-4.9, AB_3279_-4.10, and AB_3279_-4.11), three regions (AB_3279_-4.6, AB_3279_-4.12, AB_3279_-4.13) were linked to resistance under field conditions in India (PAU) and in Lebanon at KSH 2018 using a mixture of pathotypes I and II, and two genomic regions (AB_3279_-4.3, AB_3279_-4.4) were identified in a controlled environment against pathotype II and under field conditions in Ludhiana at Punjab Agricultural University (PAU) in India and in Lebanon at KSH- 2015 and KSH-2018 using a mixture of pathotypes I and II.

Two genomic regions (AB_3279_-4.1 and AB_3279_-4.2) were detected in the genotype “ILC3279” in a controlled environment against pathotype II and under field conditions in India (PAU) and in Lebanon at KSH-2015 and KSH-2018 using a mixture of pathotypes I and II. One genomic region (AB_3279_-4.5) was identified only under field conditions in India (PAU) and in Lebanon at KSH-2018 using a mixture of pathotypes I and II.

The pathotype I resistance genomic region in the genotype “ILC482” was different from the one in the genotype “ILC3279.” This conclusion is drawn from the observation, out of the eleven genomic regions controlling resistance to pathotype I in “ILC482,” seven were shared with “ILC3279,” and four genomic regions were specific to “ILC3279.” Similarly, nine genomic regions controlling resistance to the race in India (PAU) were detected in “ILC3279,” and three regions were common with “ILC482.” Four genomic regions control the resistance to pathotype I in the genotype “ILC3279” but were not identified in the genotype “ILC482.”

### Gene-Based Single Nucleotide Polymorphism Marker Associated With Resistance to *Ascochyta* Blight

In total, 21 different genomic regions associated with AB resistance were identified in CalG02 and CalG04 in both populations AB_3279_ and AB_482_. These regions contained 1,118 SNPs significantly (*p* < 0.001) associated with AB resistance, were annotated using gene ontology (GO) terms, and assigned to 319 genes (Supplementary Table 3). It was seen that the GO terms for these SNPs were uniformly assigned to each of the molecular function, biological process, and cellular component categories.

Among several genes located in the QTL regions identified in both populations were leucine-rich repeat (LRR), LEAF RUST 10 DISEASE-RESISTANCE LOCUS RECEPTOR-LIKE PROTEIN KINASE-like (LRK 10-like), and lysin motif (LysM) belonging to the family of receptor-like kinases (RLKs). Candidate genes involved in cell signaling transcription are known to regulate many cellular responses. In this study, genes encoding serine/threonine-protein kinase and protein kinase, and aspartic acid proteinase were identified and present in both populations. Also, the endoplasmic reticulum (ER) membrane and the gene encoding for protein glucan endo-1, 3- beta-glucosidase involved in disease resistance were identified.

A significant number of genes mainly involved in defense response and ethylene-mediated (ET) signaling pathways were identified, along with genes frequently responsive to ethylene mediation, like peroxidase protein. Some genes involved in stress responses like aquaporins were found in the identified genomic regions. The putative candidate genes for transcription factors (TF) WRKY and NAC were also identified in both populations.

Several repeat protein gene families have been identified in the QTL region regulating the resistance against AB including armadillo (ARM), ankyrin (ANK), HEAT, Kelch-like repeats, tetratricopeptide (TPR), leucine-rich repeats (LRRs), WD40, and pentatricopeptide repeats (PPRs), and for the first time, the gene controlling the protein GIGANTEA is found in regions associated with AB resistance in chickpea challenging AB. Also, in this study genes involved in synthesis of plant hormones such as auxin and gibberellic acids were identified and can play important roles in defense signaling against AB.

Our study shows the importance of tRNA metabolism as a point of control regulating adaptation to biotic stress, since in the genomic region associated with AB resistance, we identified genes like tRNA-Ala, tRNA-Arg, tRNA-Asn, tRNA-Gln, and tRNA-Met. Two genes encoding heat shock proteins and chaperone proteins were also identified in the QTL regions associated with AB resistance.

**FIGURE 6:**
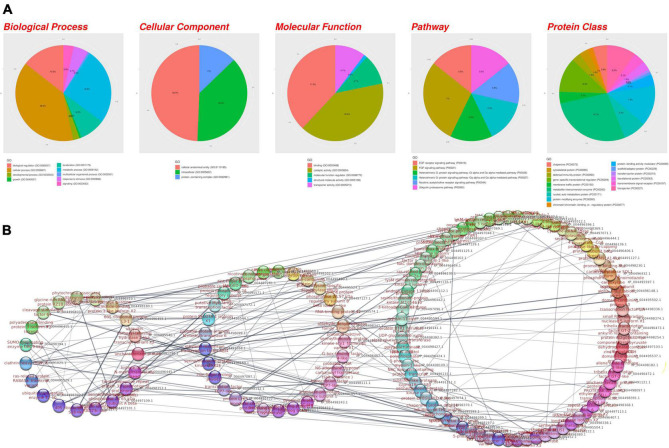
**(A)** Gene Ontology analysis and **(B)** the protein-protein interaction network of potential candidate genes for AB resistance underlying the regions identified in the AB_3279_ and AB_482_ populations. The genes are grouped by their interaction activity: high, medium, or low.

The GEA indicated that several biological pathways, such as cellular anatomical entity, catalytic activity, binding, metabolite interconversion enzyme, and protein-containing complex, are linked to AB-associated genes. Furthermore, plant defense mechanisms such as the ubiquitin proteasome pathway, defense/immunity protein, growth, chaperone, and FGF signaling pathway were found (Figure 6A).

A PPI analysis was performed to evaluate the AB-associated genes based on their biological activity and showed that the genes were related to RNA binding and regulation pathways such as 60S ribosomal protein L31 (*RPL31*). Additionally, the genes were related to plant resistance such as clathrin heavy chain2 (*CHC2*), polyadenylate-binding protein 3 (*PABPC3*), and Ras-related protein Rab-7a (*RAB7A*). Moreover, phytochrome-associated serine/threonine-protein phosphatase 1 (FYPP1) was highly interactive (Figure 6B). Furthermore, AB-associated genes linked to the mRNA surveillance pathway made a significant contribution to the gene set under consideration.

## Discussion

### Resistance to *Ascochyta* Blight in the Two Populations, AB_3279_ and AB_482_

In this study, the environmental conditions, especially temperature, have important effects on the development of *Ascochyta rabiei* and infection process. At Terbol station in Lebanon and in 2015, AB did not develop well because of low temperature following inoculation. At the Kfarshakhna station, AB infection was relatively higher in 2015/16 than in 2018/19, which could be explained by exposure to chilling temperatures (Kemal et al., 2017). At KSH in the 2018 season, it appears that the environment conditions were more conducive to the development of pathotype II than pathotype I.

In the greenhouse experiments, the inoculation was successful and showed that “ILC482” is highly susceptible to pathotype II and resistant to pathotype I, thereby confirming the results of Imtiaz et al. (2011) and Kemal et al. (2017). Most known *A. rabiei*-resistant sources used in breeding programs have non-complete/incomplete resistance, and moderate resistance has been identified in several chickpea accessions (Sharma and Ghosh, 2016). In this study, “ILC3279” exhibited a resistant to moderately resistant reaction, and “ILC482” exhibited a resistant to susceptible reaction under controlled and field conditions against pathotypes I and II, and the race from India.

In the WANA region,”ILC3279” and “ILC482” have shown resistant to moderately resistant reactions to AB. Using different genotypes, “ILC3279” is resistant to pathotypes I and II, while “ILC482” is resistant to pathotype I and susceptible to pathotype II (Udupa and Baum, 2003; Imtiaz et al., 2011; Kemal et al., 2017). Labdi et al. (2013) reported that the resistance in “ILC3279” was controlled either by three recessive genes or two recessive duplicated genes. Singh et al. (1984) found that “ILC482” resistance is governed by an independent dominant gene.

Evaluation of the two RIL populations for disease score under controlled and field conditions indicated considerable variation among the lines for their reaction to AB. The reaction of the two RIL populations (AB_3279_ and AB_482_) showed transgressive segregation for AB resistance in all locations and the seedling stage in a greenhouse. This transgressive segregation was also reported by Sabbavarapu et al. (2013) for AB resistance in the seedling stage in a controlled environment facility (CEF) at ICRISAT- Patancheru using the same source of resistant “ILC3279.” Sabbavarapu et al. (2013) found a normal distribution of the reaction in the APR stage in Ludhiana at Punjab Agricultural University in 2013 using the same race in the F2 population issued from the same source of resistant “ILC3279” crossed with a different susceptible parent, “C214.”

Significant effects of genotypes, sites, year, interactions of genotype and site, and year were detected for AB reaction, which could be attributed to differences in total precipitation during the growing seasons (Table 2), as also reported also by Kemal et al. (2017), or to differences in virulence spectra of isolates and populations of the pathogen (Purcell, 2002).

High broad-sense heritability was found for AB reaction in both populations AB_3279_ (H^2^ = 0.62–0.87) and AB_482_ (H^2^ = 0.58–0.84) under controlled and field conditions. Sabbavarapu et al. (2013) have also reported high heritability (0.78) using the same resource of resistant “ILC3279.” Moderate to high broad-sense heritability was reported under controlled and field conditions (0.48–0.65) by Deokar et al. (2018). However, other studies have reported lower broad-sense heritability, such as 0.36 by Cobos et al. (2006), 0.38–0.54 by Lichtenzveig et al. (2006), and 0.14–0.34 by Daba et al. (2016).

Transgressive segregation and moderate to high heritability call for identification of additional sources of resistance and their use in multiple crosses to accumulate more genes and achieve an acceptable and stable level of resistance to AB. The use of molecular techniques and QTLs associated with resistance could help achieve this goal.

### Quantitative Trait Loci and Genomic Regions Associated With Resistance to *Ascochyta* Blight in the AB_3279_ (ILC3279 × ILC1929) and AB_482_ (ILC482 × ILC1929) Populations

In this study, all the QTLs associated with AB resistance are located in CaLG02 (chromosome 2) and CaLG04 (chromosome 4) in both populations, confirming the results by other studies conducted in different environments in the Middle East and India, and for different plant stages (Cho et al., 2004; Sharma and Ghosh, 2016).

In a previous study, some QTLs and genomic regions have been identified in populations derived from crosses between resistant genotype “ILC3279” and several different susceptible parents (Supplementary Table 2). However, all these QTLs were identified using low-density SSR marker-based maps, and QTL interval varied from 0.3 to 30 Mb in “ILC3279.” This study represents the first report on genomic analysis for AB resistance in the genotype “ILC482.” We have identified 21 genomic regions associated with AB resistance in CaLG02 and CaLG04 in chickpea (Supplementary Table 1). The proposed physical map narrowed down the regions that confer AB resistance, with consistent genomic regions identified across different environments.

Four major genomic regions in CalG02 regulating AB resistance in the genotype “ILC3279,” AB_3279_-2.1, AB_3279_-2.2, AB_3279_-2.3, and AB_3279_-2.4, were identified in different environments against different pathotypes and populations. Physical mapping of previously reported QTLs in CaLG02 (Supplementary Tables 1, 2) for resistance to AB in the cultivar “ILC3279” using flanking SSR marker sequences revealed QTLs near the marker GA16-indicative QTL *ar1*, *ar2a* located at 34747499 Mb (Udupa and Baum, 2003). However, our study suggested that the QTL *ar1, ar2a* could be different than the four regions, with the marker GA16 at a distance of 2.3 Mb from the closest region in our study. Similarly, a different region from *QTLAR3* appeared tightly linked with STMS TA194, and flanked marker TR58-TS82, located at TR58: 29252945 Mb, was identified by Iruela et al. (2007). Varshney et al. (2014a) identified a region flanked by markers GA16-TS82 and TA194 located at GA16: 34747740, which is different from regions detected in CaLG02 in the cultivar “ILC3279” in our study. The markers TS82 and TA194 were not aligned to the chromosomes in this study. TS82 was physically mapped at 25798007 bp in CaLG04, while the marker TA194 was not aligned to any of the chickpea chromosomes in this study.

The four regions detected in this study in CaLG02 overlapped with previous QTLs that were detected in other different sources of resistance (FLIP84-92C) like *QTLAr19* flanked by marker TA37-GA16 located at (17196574–34747740 Mb ∼17.6) (Cho et al., 2004). The findings also suggested that these four regions were distinct from other QTLs *QTLAr21d* flanked by TA37–TA200 markers at 171196574–15459886 (Cho et al., 2004). Similarly, the four regions overlapped with *QTL1* flanked by marker TR19-TA110 physically located at 27187236–9410378 Mb ∼17.8 (Anbessa et al., 2009) using different genotypes (CDC Luna). Our study allowed us to narrow down the region from 17.6 and 17.8 Mb, respectively, reported by Cho et al. (2004) and Anbessa et al. (2009) studies to four regions, AB_3279_-2.1 (95 kb), AB_3279_-2.2 (32 bp), AB_3279_-2.3 (55 kb), and AB_3279_-2.4 (306 kb).

The four regions were different from the candidate gene *Ein3* linked to *QTLAR3* located at 32865451–32867779 Mb identified in a different source of resistant “ILC72” (Madrid et al., 2014). The majority of these QTLs was identified using low-density genetic maps; hence, the QTLs were mapped in large genomic intervals containing hundreds of potential candidate genes. More recently, Deokar et al. (2019) reported QTLs for AB resistance in CaLG02 using the GBS approach, but we are not able to compare if they are the same QTLs as in our study because of the unavailability of CDC Frontier reference genome assembly v2.6 online.

In this study, we did not map any QTLs linked with resistance to pathotype II in CaLG02 under controlled and field conditions, while Udupa and Baum (2003) reported that *QTLar2a* was detected in CaLG02 for resistance to pathotype II, and that both pathotypes I- and II-specific loci in CaLG02 were tightly linked. No QTLs or regions regulating AB resistance were identified in CalG02 in “ILC482” in different environments and against different races and pathotypes.

As part of this study, 13 genomic regions in the AB_3279_ population and 11 genomic regions in the AB_482_ population were associated with AB resistance in CalG04. Seven major genomic regions were common between the AB_3279_ and AB_482_ populations in different environments. Physical mapping of previously reported QTLs in CaLG04 for resistance to AB, using flanking SSR marker sequences, revealed many QTLs (Supplementary Tables 1, 2).

The genomic region AB_3279_-4.5 spanned 8.66 to 8.87 Mb and overlapped with a QTL flanked by GA24-GAA47 (9.1–8.28 Mb ∼) linked to resistance to pathotype II (Cho et al., 2004). This region was narrowed from 820 to 216 kb (8660115–8875933 Mb).

Three genomic regions, AB_3279_-4.3, AB_482_-4.3, and AB_3279–482_.2 (AB_3279_-4.4 and AB_482_-4.4), overlapped with the previously reported *QTLAR1* (flanked by NCPGR91-GAA47 located at 4.58–8.28 Mb ∼ 3.7 Mb) (Madrid et al., 2012). However, this region was narrowed for AB_3279_-4.3 (60 kb), AB_482_-4.3 (200 kb), and AB_3279_-4.4 and AB_482_-4.4 (92 kb). The ethylene receptor-like sequence (*CaETR*-) gene-linked marker was shown to be associated with resistance in the *QTLAR1* region and located at 4.57–4.58 Mb (Madrid et al., 2013). However, Madrid et al. (2013) reported that the resistance allele of the *CaETR*-1 marker was absent in several chickpea AB-resistant accessions whose source of AB resistance was “ILC3279.” Also, we did not identify any SNPs or QTLs in this region in both resistant parents “ILC3279” and “ILC482.”

The genomic region AB_482_-4.6, spanning from 15.7 to 15.86 Mb–159 kb, overlapped with one of the earlier reported QTL by Li et al. (2017) who identified a 100-kb region (*AB4*.1) in CaLG04 (Ca4: 15,855,018–15,980,584) by genome-wide association studies (GWAS) on Australian chickpea accessions whose sources of AB resistance were “ICC3996,” “ICC14903,” and “ICC13729.” We used GBS to narrow down the region from 100 kbp to 714 bp in the population AB_482_.

Kumar et al. (2018) identified two major QTLs, *qABR4*.*1* (2.73–5.24 Mb) and *qABR4*.*2* (27.55–33.49 Mb), and a minor QTL, *qABR4*.3 (38.78–39.48 Mb) in CaLG04 using FLIP84-92C (2) as source of resistance. In this study, six regions, *AB*_3279–482_.1, AB_3279–482_.2, AB_3279_-4.2, AB_3279_-4.3, AB_482_-4.2, and AB_482_-4.3, overlapped with *qABR4*.*1*, but we narrowed the region from 2.51 Mb to six regions AB_3279–482_.1 (108 kb), AB_3279–482_.2 (92 kb), AB_3279_-4.2 (12.3 kb), AB_3279_-4.3 (60 kb), AB_482_-4.2 (263 kb), and AB_482_-4.3 (200 kb). Similarly, the QTL *qABR4*.3 was narrowed from 700 to 195 kb (38785205–38979793 Mb).

Kumar et al. (2018) mentioned that the genomic region under *qABR4*.2 (27.55–33.49 Mb ∼5.94 Mb) shares the SCY17_590_ marker locus, which was previously associated with *QTL_*AR*2_* (Iruela et al., 2006). Although Iruela et al. (2006) used the same source of resistance, “ILC3279”; our results suggested that *QTLAR2* and *qABR4*.2 could be different than the regions detected in “ILC3279” and “ILC482,” suggesting that this particular region of CaLG04 requires more efforts for better assembly because of the presence of many QTLs and regions regulating AB resistance in different sources of resistance and against different pathotypes and races. We confirmed that *qABR4*.2 and *QTL_*AR*2_* are different from regions in the neighborhood of the SCY17_590_ marker locus that explains the highest AB resistance in QTL*_*AR*2_* (Iruela et al., 2006). Also, the *qABR4*.2 region harbors the *CaAHL21* gene (LOC101509190, which spanned from 29156785 to 29157696 Mb), and region or SNPs regulating AB resistance were identified in both populations.

Kumar et al. (2018) narrowed *qABR4*.1 to a “robust region” at 4.568–4.618 Mb, and this study narrowed further this region from 50 to 12.3 kb (4605334–4617641 Mb) located in the region AB_482_-4.3. Kumar et al. (2018) showed the CaAHL18 gene is the candidate gene under “robust qABR4.1.” Earlier, CaETR-1’s polymorphic marker was shown to be associated with AB resistance in chickpea (Madrid et al., 2012), and Kumar et al. (2018) reported that *CaETR*-1 is flanked on both sides by *CaAHL17*, which spanned from 4569510 to 4574907, and *CaAHL18, which* spanned from 4593474 to 4594948, both having polymorphism between resistant and susceptible parents. The regions identified by Madrid et al. (2012) and Kumar et al. (2018) may be different from the ones identified in our study because of the use of different pathotypes of AB and different sources of resistance. The gene CaAHL18 was not identified in our study. Kumar et al. (2018) validated the marker CaNIP8 in 24 different accessions of chickpea, among them ILC3279 and ILC482; but in our study, we did not identify any QTL or region in the vicinity of the CaNIP8 marker.

Recently, Deokar et al. (2018) reported QTLs for AB resistance with CaLG02 (NGS)-based BSA approach, but we were not able to compare if they were the same QTLs as in our study because of unavailable CDC Frontier reference genome assembly v2.6 online.

### Pathotype-Specific Regions for *Ascochyta* Blight Resistance

Despite the presence of different pathotypes, many genetic studies have ignored the pathotype-specific effects of QTLs, and only a few have mentioned the AB pathotype in their genetic studies (Udupa and Baum, 2003; Cho et al., 2004; Aryamanesh et al., 2010; Li et al., 2017; Kumar et al., 2018).

The genetic nature for pathotype-specific resistance to AB in the two RIL populations AB_3279_ and AB_482_ showed significantly different resistance patterns to pathotypes I and II. A previous study has reported that the genotype “ILC3279” was resistant to pathotypes I and II, and that the genotype “ILC482” was only resistant to pathotype I (Imtiaz et al., 2011).

The pathotype I-resistant genomic region in the genotype “ILC482” is different from the one in the genotype “ILC3279.” This conclusion is drawn from the observation that out of the eleven genomic regions controlling the resistance to pathotype I in “ILC482,” seven were shared with “ILC3279,” and four genomic regions were specific for “ILC3279.” Similarly, nine genomic regions controlling the resistance to the race from India at Punjab Agricultural University (PAU) in Ludhiana were detected in “ILC3279” and three were common with “ILC482.”

Udupa and Baum (2003) reported in their study that resistance to pathotype I is controlled by a major locus (*ar2a* in CaLG02) with flanking marker GA16 (347474499), and that resistance to pathotype II is controlled by two recessive loci with complementary gene action, *ar2a* in CaLG02 with flanking marker GA16 (347474499) and *ar2b* in CaLG04 with flanking markers TA130-TA72 and TS72 (TS72: 40036747), using “ILC3279” as source of resistance. Cho et al. (2004) concluded that a locus (*Ar19*) in CaLG02 is linked to resistance to pathotype I, with flanking marker GA20-GA16 (34747581–34747499), and that a QTL in CaLG04 is the major locus for resistance to pathotype II, with flanking marker GA24-GAA47 (8284223–9101902 Mb) in “FLIP84-92C.” In the same study, Cho et al. (2004) reported that *Ar19* controls resistance to pathotype II by additive interaction with the QTL in LG4A. Our findings did not detect any QTL or region associated with resistance to pathotype II in CaLG02, and the four genomic regions AB_3279_-4.1 (30902858–30997784 Mb), AB_3279_-4.2 (32108597–32108629 Mb), AB_3279_-4.3 (32109805–32415818 Mb), and AB_3279_-4.4 (32432904–32488272 Mb) were detected in the genotype “ILC3279” in a controlled environment against pathotype I; those QTLs were new and different from previously reported QTLs.

The four genomic regions detected in CaLG04 associated with resistance to pathotype II in “ILC3279,” AB_3279_-4.1 (3990334–4098404 Mb), AB_3279_-4.2 (4141107–4153455 Mb), AB_3279_-4.3 (4811896–4872102 Mb), and AB_3279_-4.4 (4882698–4974830 Mb), were different from regions detected in previous studies (Udupa and Baum, 2003; Cho et al., 2004).

Li et al. (2017) reported a 100-Kb region (AB4.1) in CaLG04 (Ca4: 15,855,018–15,980, 584) using ICC3996, ICC14903, and ICC13729 against an isolate belonging to pathotype IV, and our study identified AB_482_-4.6 from 15.7 to 15.86 Mb in “ILC482” in a controlled environment against pathotype I and narrowed the region to 5.2 kb (15855018–15860311 Mb).

Kumar et al. (2018) identified two major QTLs, *qABR4*.*1* (2.73–5.24 Mb) and *qABR4*.*2* (27.55–33.49 Mb), and a minor QTL, *qABR4*.3 (38.78–39.48 Mb) in CaLG04 using FLIP84-92C (2) as source of resistance against an isolate belonging to pathotype II. The four regions associated with resistance to pathotype II in our study using “ILC3279” overlapped with *qABR4*.*1*, and one major region, AB_3279–482_.7, was identified in a controlled environment against pathotype I in “ILC3279” and “ILC482” and under field conditions in Ludhiana at Punjab Agricultural University (PAU) India and Lebanon at KSH-2015 and KSH-2018 using a mixture of pathotypes I and II in “ILC3279,” but no QTL with resistance to pathotype II was identified.

All alleles in the AB-resistant loci showed negative additive effects originating from “ILC1929,” which indicates that the increase in resistance is due to the absence of alleles from the moderately resistant parent “ILC3279,” and that the recessive resistant alleles were concentrated in “ILC3279.” Notably, the presence of a host factor in the specific region in ILC3279 confers the recessive gene. Udupa and Baum (2003) also reported that the resistance gene is recessive in “ILC3279.” Similarly, Hashimoto et al. (2016) reported that resistance is conferred by a recessive gene mutation that encodes a host factor critical for viral infection. It is a branch of the resistance machinery and, as an inherited characteristic, is very durable.

### Gene-Based Single Nucleotide Polymorphism Markers Associated With Resistance to *Ascochyta* Blight

In total, 21 different genomic regions were identified in CalG02 and CalG04 pertaining to AB resistance in both populations AB_3279_ and AB_482_. These regions contain 319 genes (Supplementary Table 3). Among these genes, several have been previously reported as key factors for pathogen resistance in different plant species. For instance, we have located genes such as *F-box, RLK*, and *GIGANTEA* located near or adjoining AB-resistance associated SNPs. The *F-box* protein mediating protein ubiquitination and degradation was shown to be involved in plant defense and plays important roles in stress responses and disease resistance (Jia et al., 2017; Zhang et al., 2019). In our study, genes encoding for serine/threonine-protein kinase or protein kinase and aspartic acid proteinase were identified in both populations. Few repeat receptor-like kinases (*RLK*) genes are known to play essential roles mainly in plant defense and one of the major groups is *LysM-RLK*, which plays a critical role in fungal resistance by perception of the fungal cell wall component in Arabidopsis (Jose et al., 2020).

Genes linked to auxin and gibberellic acids were identified in the AB-associated gene, which could play an important role in defense signaling against AB. Garg et al. (2019) reported the presence of some plant hormone genes in resistant chickpea genotypes against AB under controlled environments. Amorim et al. (2017) reported the role of three families of transcription factors, *ERF*, *bZIP*, and *WRKY*, and suggested that they are important players in response to biotic stresses such as insect attack and pathogen infection. Similarly, Garg et al. (2019) reported the presence of TFs like *bHLH*, *WRKY*, and *MYB* in resistant chickpea genotypes compared to genotypes susceptible to AB.

Additionally, genes correlated with flowering time and regulated transcription of several floral integrator genes were detected. Genes linked to plant flowering were reported in *Fusarium oxysporum* resistance (Lyons et al., 2015). Some of the genes detected include *GIGANTEA*, which promotes flowering time and enhances susceptibility to infection and pathogen defense (Ridge et al., 2017). The possibility of the presence of many genes in each region and its associated proteins having a role in AB resistance can truly fulfill the conclusions derived from earlier genetic studies that two major complementary genes along with several modulators are involved in AB resistance of chickpea (Tekeoglu et al., 2002; Iruela et al., 2006).

We performed GEA and PPI analyses on potential candidate genes linked to AB resistance in the AB_3279_ and AB_482_ populations (Figures 6A,B). Genes associated with disease resistance pathways, such as immunity protein, growth, and chaperone pathways, are abundant in the detected AB-associated genes. The activity of chaperone is linked to several important mechanisms in plants; majority of which are related to environmental stress (Bulgakov et al., 2019) such as heat shock (Wei et al., 2021) and disease (Islam et al., 2020).

A PPI analysis was performed to evaluate AB-resistant genes based on their interaction activity in the biological system. The PPI analysis showed that genes related to plant resistance such as clathrin heavy chain2 (*CHC2*) (Wu et al., 2015), polyadenylate-binding protein 3 (*PABPC3*) (Yang and Hunt, 1994), Ras-related protein *Rab*-7a (*RAB7A*), and phytochrome-associated *serine*/*threonine*-protein phosphatase 1 (*FYPP1*) are highly interactive (Figure 1B). Genes associated with the mRNA surveillance pathway contributed significantly to the reported AB-associated gene set. This pathway contains plant genes that detect and degrade abnormal mRNAs, such as those produced by viral infections (Kawa, 2020).

## Conclusion

This study provides a clear understanding of the quantitative nature of resistance to *Ascochyta* blight in two populations of chickpea. We report a GBS-based high-density consensus linkage map with a potential to facilitate efficient anchoring of QTLs to a physical map. This study provides tightly linked SNP markers for marker-assisted selection of *Ascochyta* blight resistance in chickpea. The GBS-SNP markers enabled automation and high-throughput genotyping and consistent QTLs linked to the desirable traits. The proposed physical map narrowed down AB resistance regions, with consistent genomic regions identified across different environments, which will help in breeding programs for chickpea improvement.

## Data Availability Statement

The original contributions presented in the study are publicly available in Zenodo: https://doi.org/10.5281/zenodo.5236818.

## Author Contributions

AAm: supervisor, fund, and manuscript review. ARR: supervisor. MB: manuscript review. ZK: fund. SU: RIL development and manuscript review. KA-S: phenotype data analysis. AMA: R script. SS: fund and manuscript review. TI, UR, and BA: filed work and disease screening. AH: plant collection. All authors contributed to the article and approved the submitted version.

## Conflict of Interest

The authors declare that the research was conducted in the absence of any commercial or financial relationships that could be construed as a potential conflict of interest.

## Publisher’s Note

All claims expressed in this article are solely those of the authors and do not necessarily represent those of their affiliated organizations, or those of the publisher, the editors and the reviewers. Any product that may be evaluated in this article, or claim that may be made by its manufacturer, is not guaranteed or endorsed by the publisher.
