# Supplementary material for: Novel Genomic Regions Linked to Ascochyta Blight Resistance in Two Differentially Resistant Cultivars of Chickpea
Source: Front Plant Sci. 2022 Apr 25;13:762002. doi: 10.3389/fpls.2022.762002 (PMC9083910; doi:10.3389/fpls.2022.762002)
Supplement: Supplementary file 1 [file Data_Sheet_2.docx]

**Supplementary S1: Field screening**

The seeds were planted in 12 blocks, 1 row/line, 20 seeds/row with planting distance/row of 10 cm, row length 200 cm, and distance between adjacent rows 45 cm. Susceptible checks ‘ILC263’, ‘FLIP 11-1C’ (Sel74102) were used. The distribution of susceptible checks in the field was after every 20 rows of test genotypes. The plant lifecycle stage at the first inoculation was 4-6 leaves (seedling stage). The inoculations were done in the evening when mild temperature (18-22°C) conditions prevailed. The field was irrigated before inoculation. From 7 days old colonies of each pathotype, first spore suspension concentration was adjusted to 5 x 10^5^ ml^-1^ using cell counter then chickpea plants were inoculated using motorized sprayer. The epiphytotic conditions were created with the help of a perfo-sprayer system to maintain the relative humidity beyond 85% and temperature around 25°C. Mild temperature (18-22°C) and high relative humidity (85-95%) are congenial for the quick development of the disease. The perfo-sprayer system was run during daytime from 10:00-16:00 hrs for 21 days at an interval of one hour. The disease symptoms started appearing after 10-15 days of inoculation. Observations were recorded on 1-9 rating scale in the last week of March of the season at which time the plants were at the pre-flowering stage (Singh and Sharma 1998; Gurha*et al*. 2003).

The same field screening technique was adopted for evaluation of the material at Punjab Agricultural University, Ludhiana, Punjab, India. The material was planted in 200 cm rows spaced at 40 cm apart. Susceptible check ‘JG 62/L550’ was planted as indicator-cum-infector rows after every 8 test rows. The field was irrigated in the morning on the day of inoculation. For artificial inoculation, 7-10 days old culture multiplied on broth was used. The spore count of the inoculum was adjusted to 4x10^4^ spores ml^-1^ with a haemocytometer. Inoculation of all the test and check entries was done by spraying spore suspension in the evening 18:00 hr in the first week of February (the crop was in flower initiation stage i.e. approximately 85-90 days after sowing) with motorized sprayer for uniform spread and proper establishment of the disease. Relative humidity of 85% was maintained by sprinkling water with perfo-spray system during the daytime for 10-15 minutes at 1.5-2.0 h interval (depending on the prevailing environmental conditions) from 10.00 to 16.00 h for 21 days. The disease symptoms started appearing 10-15 days after inoculation period.


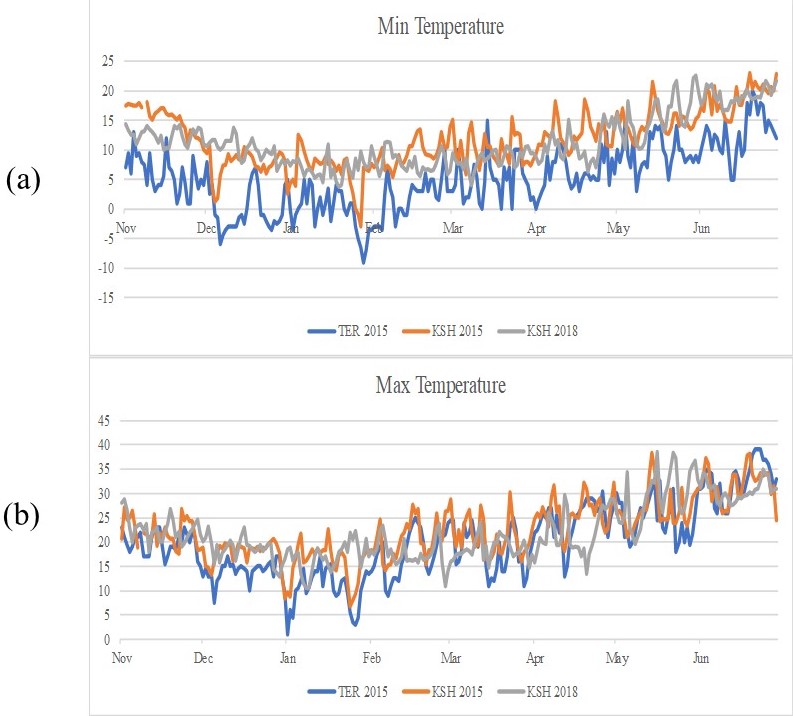


**Figure 1: Minimum temperature and Maximum temperature during cropping seasons.** (a) Minimum temperature and (b) Maximum temperature November to June during the growing season at Kfarshakhna 2015-2016 and 2018-2019, and Terbol in 2015-2016 (Lebanon)

**CaLG02**

**Supplementary Figure 2: High density intra-specific genetic map of chickpea (ILC3279 × ILC1929) using GBS-SNP markers. Linkage** groups 2. Genetic distances (cM) were shown on the left side and the markers were shown on the right side of the bars. Map was constructed using JoinMap 4.0 and Kosambi mapping function. Markers in black colour font are from the framework map and markers in red colour font are QTLs.

| **CaLG04 A**  **** | **CaLG04 B**  **** | **CaLG04 C**  **** | **CaLG04 D**  **** |
| --- | --- | --- | --- |

**Supplementary Figure 3: High density intra-specific genetic map of chickpea (ILC3279 × ILC1929) using GBS-SNP markers.** Linkage groups 4. Genetic distances (cM) were shown on the left side and the markers were shown on the right side of the bars. Map was constructed using JoinMap 4.0 and Kosambi mapping function. Markers in black colour font are from the framework map and markers in red colour font are QTLs. For clear visualization, CaLG04 were split into four parts and named as CaLG04A, CaLG04B, CaLG04C and CaLG04D.

AB_3279_-LG4-Hotspot-1

| **CaLG04 A**  **** | **CaLG04 B**  **** | **CaLG04 C**  **** | **CaLG04 D**  **** |
| --- | --- | --- | --- |

**Supplementary Figure 4: High density intra-specific genetic map of chickpea (ILC482 × ILC1929) using GBS-SNP markers.** Linkage groups 4. Genetic distances (cM) were shown on the left side and the markers were shown on the right side of the bars. Map was constructed using JoinMap 4.0 and Kosambi mapping function. Markers in black colour font are from the framework map and markers in red colour font are QTLs. For clear visualization, CaLG04 were split into four parts and named as CaLG04A, CaLG04B, CaLG04C and CaLG04D.

**Supplementary Table 1: comparison of Genomic regions associated with AB resistance in chickpea among different studies.**

| **Chromosome** | **Region** | **Associated populations** | **Genomic location bp** | **References** |
| --- | --- | --- | --- | --- |
| **CaLG02** | AB_3279_-2.1 | AB_3279_ | 30902858-30997784 ~ 95 kb | Cho et al (2004) and (Anbessa *et al*. 2009) |
|  | AB_3279_-2.2 | AB_3279_ | 32108597-32108629 ~ 32 pb | Cho et al (2004) and (Anbessa *et al*. 2009) |
|  | AB_3279_-2.3 | AB_3279_ | 32109805-32415818 ~ 306 kb | Cho et al (2004) and (Anbessa *et al*. 2009) |
|  | AB_3279_-2.4 | AB_3279_ | 32432904-32488272 ~ 55 kb | Cho et al (2004) and (Anbessa *et al*. 2009) |
| **CaLG04** | AB_3279-482_.1 | AB_3279_, AB_482_ | 3990334-4098404 ~ 108 Kb | Kumar *et al*. (2018) |
|  | AB_3279-482_.2 | AB_3279_, AB_482_ | 4882803-4974830 ~ 92 Kb | Madrid *et al*. (2012), Kumar *et al*. (2018) |
|  | AB_3279-482_.3 | AB_3279_, AB_482_ | 10931884-11771922 ~ 840 Kb | The present study |
|  | AB_3279-482_.4 | AB_3279_, AB_482_ | 15933422-16076690 ~ 143 Kb | The present study |
|  | AB_3279-482_.5 | AB_3279_, AB_482_ | 16990705-17037384 ~ 47 Kb | The present study |
|  | AB_3279-482_.6 | AB_3279_, AB_482_ | 38008706-38979508~ 970 Kb | The present study |
|  | AB_3279-482_.7 | AB_3279_, AB_482_ | 37348105-37818047 ~ 470 Kb | The present study |
|  | AB_3279_-4.2 | AB_3279_ | 4141107-**4153455** ~ 12.3 Kb | Kumar *et al*. (2018) |
|  | AB_3279_-4.3 | AB_3279_ | 4811896-4872102 ~ 60 Kb | Madrid *et al*. (2012), Kumar *et al*. (2018) |
|  | AB_3279_-4.5 | AB_3279_ | 8660115-8875933 ~ 216 Kb | Cho *et al.* (2004) |
|  | AB_3279_-4.9 | AB_3279_ | 34784962- 35664917~ 880 Kb | The present study |
|  | AB_3279_-4.10 | AB_3279_ | 36368625-36983721~ 615 Kb | The present study |
|  | AB_3279_-4.11 | AB_3279_ | 37160576-37293408 ~ 133 Kb | The present study |
|  | AB_482_-4.2 | AB_482_ | 4205361-4468395 ~ 263 Kb | Kumar *et al*. (2018) |
|  | AB_482_-4.3 | AB_482_ | 4605334-4805677 ~ 200 Kb | Madrid *et al*. (2012), Kumar *et al*. (2018) |
|  | AB_482_-4.6 | AB_482_ | 15701299-15860311 ~ 159 Kb | Li *et al*. (2017) |
|  | AB_482_-4.8 | AB_482 (55)_ | 16127324-16865990 ~ 739 Kb | The present study |
|  | | | | |

**Supplementary Table 2: Genomic location of previously identified QTLs for Ascochyta blight resistance in chickpea.**

| **Chromosome** | | **QTL** | **QTL Lined marker** | | | **Genomic location bp** | | | **References** | |  |  |  |  |  |
| --- | --- | --- | --- | --- | --- | --- | --- | --- | --- | --- | --- | --- | --- | --- | --- |
| **CaLG02** | | Ar1, ar2a | GA16 | | | 34747740 | | Udupa and Baum (2003) | | |  |  |  |  |  |
|  | | Ar19 | TA37-GA16 | | | 17196574-34747740 | | Cho *et al.* (2004) | | |  |  |  |  |  |
|  | | Ar21d | TA200-TA37 | | | 15459886-17196574 | |  | | |  |  |  |  |  |
|  | | QTLAR3 | TR58-TS82 | | | 29252945- 25798007 | | Iruela *et al.* (2007) | | |  |  |  |  |  |
|  | | QTL1 | TA110-TR19 | | | 9410378-27187236 | | Anbessa *et al.* (2009) | | |  |  |  |  |  |
|  | | Ein3 | GA16-TA194 | | | 32865451-32867779 | | Madrid *et al.* (2014) | | |  |  |  |  |  |
|  | | ABQTL-I | GA16, TA194, TS82 | | | 32865451 | | Varsheny *et al.* (2014) | | |  |  |  |  |  |
| **CaLG04** | | QTLAR2 | TR20-TA72 | | | 23273185-43563684 | | Udupa *et al.* (2003) | | |  |  |  |  |  |
|  | | QTLAR1 | GA24-GAA47 | | | 8006877-8802468 | | Cho *et al.* (2004) | | |  |  |  |  |  |
|  | | QTLAR2 | TA146-TA72 | | | 24367557-43563684 | | Iruela *et al.* (2006) | | |  |  |  |  |  |
|  | | QTLAR1 | NCPGR91-GAA47 | | | 4411370-8006877 | | Madrid *et al.* (2012) | | |  |  |  |  |  |
|  | | QTLAR1 | CaETR-1 (LOC101508648) | | | 4411499 | | Madrid *et al.* (2013) | | |  |  |  |  |  |
|  | | QTLAR1 | CaSTMS11-TA130 | | | 8802388-15658926 | | Sabbavarapu *et al.* (2013) | | |  |  |  |  |  |
|  | | ABQTL-II | GAA47, SCY17, TA130, TA2 | | | 8802468 | | Varsheny *et al.* (2014) | | |  |  |  |  |  |
|  | | AB4.1 | SNP15855018-SNP15980584 | | | 15855018-15980584 | | Li *et al.* (2017) | | |  |  |  |  |  |
|  | | QTLAR1 | CaNIP18-CaNIP12, | | | 4314671-4506563 | | Kumar *et al.* 2018 | | |  |  |  |  |  |
|  | | | | | | | | | | |  |  |  |  |  |
|  |  | |  | | |  |  | |  | | |  | | SSR- 3 | Varsheny*et al.* (2014) |

**Supplementary Table 3: List of potential candidate genes for Ascochyta blight resistance underlying the regions identified in AB_3279_ and AB_482_ populations.**

|  | Gene ID | Position | Name |
| --- | --- | --- | --- |
| **CalG02** | LOC101493700 | 32109496-32114858 | TMV resistance protein N-like |
|  | LOC101514321 | 32119728-32122703 | glucan endo-1,3-beta-glucosidase 12-like |
|  | LOC101514856 | 32122703-32127909 | small ubiquitin-related modifier 1-like |
|  | LOC101489157 | 32139549-32148033 | subtilisin-like protease Glyma18g48580 |
|  | LOC101489486 | 32155262-32158391 | MDIS1-interacting receptor like kinase 2-like |
|  | LOC101489804 | 32169657-32173671 | endoglucanase 11-like |
|  | LOC113785508 | 32184589-32199085 | cucumisin-like |
|  | LOC101490875 | 32201586-32204785 | cucumisin-like |
|  | LOC101494336 | 32225391-32226035 | Uncharacterized protein |
|  | LOC101494542 | 32235179-32239755 | Uncharacterized protein |
|  | LOC101494861 | 32241740-32244161 | protein LYK5 |
|  | LOC101495186 | 32246933-32250559 | protein NRT1/ PTR FAMILY 6.4 |
|  | LOC101495511 | 32251196-32252060 | protein DMP3-like |
|  | LOC101495840 | 32256721-32261177 | homeobox protein SBH1-like isoform |
|  | LOC101496165 | 32274347-32278609 | plant UBX domain-containing protein 4-like |
|  | LOC101496494 | 32278939-32289068 | protein FAM91A1-like, |
|  | LOC101496828 | 32295662-32300359 | receptor-like protein EIX2 isoform |
|  | LOC101497482 | 32335071-32338948 | receptor-like protein EIX1 isoform |
|  | LOC101498012 | 32347030-32353238 | Uncharacterized protein |
|  | LOC101491819 | 32375031-32378826 | exosome complex component RRP40-like |
|  | LOC101498343 | 32386805-32389841 | Uncharacterized protein |
|  | LOC101499009 | 32407079-32412077 | lysM domain receptor-like kinase 3 isoform |
|  | LOC101499326 | 32414603-32418973 | lysM domain receptor-like kinase 3 isoform |
|  | LOC101492147 | 32419667-32422175 | UPF0481 protein At3g47200-like |
|  | LOC101492489 | 32422864-32427720 | protein MAIN-LIKE 1-like |
|  | LOC101492821 | 32431204-32435017 | uncharacterized protein |
|  | LOC101493150 | 32439678-32442039 | receptor-like protein 9DC3 isoform |
|  | LOC105851670 | 32473279-32476267 | receptor-like protein 53 |
|  | LOC101499643 | 32481196-32487415 | CSC1-like protein At3g21620 |
| **CalG04** | LOC101499129 | 30902305-30933427 | anaphase-promoting complex subunit 1 |
|  | LOC101514746 | 30944203-30957587 | clathrin heavy chain 2 |
|  | LOC101514420 | 30959566-30960992 | transcription factor MYB44-like |
|  | LOC101514211 | 30965426-30966024 | uncharacterized |
|  | LOC101513864 | 30980818-30981180 | RING-H2 finger protein ATL66 |
|  | LOC101499450 | 30997204-30998536 | ethylene-responsive transcription factor ABI4 |
|  | LOC101513679 | 3990027-3991089 | probable aquaporin TIP-type alpha |
|  | LOC101514344 | 3994879-3996463 | NDR1/HIN1-like protein 6 |
|  | LOC101514671 | 3997037-3999457 | coiled-coil domain-containing protein 12 |
|  | LOC101489067 | 4027853-4029479 | dof zinc finger protein DOF2.5-like |
|  | LOC101509177 | 4047101-4051761 | transcription factor bHLH157 |
|  | LOC101490379 | 4094002-4098898 | plant UBX domain-containing protein 10 |
|  | LOC101509506 | 4100727-4102465 | protein yippee-like At4g27745 |
|  | LOC101490702 | 4103056-4104110 | mavicyanin |
|  | LOC101491021 | 4105195-4115232 | serine/threonine-protein kinase BLUS1, transcript |
|  | LOC101491850 | 4142360-4149419 | protein HLB1 |
|  | LOC101493821 | 4217658-4229165 | ubiquitin carboxyl-terminal hydrolase 9-like isoform |
|  | LOC101494148 | 4230279-4234860 | glucan endo-1,3-beta-glucosidase 11 |
|  | LOC101495532 | 4251209-4258964 | nuclear pore complex protein NUP98A isoform X7 |
|  | LOC101497390 | 4275782-4285588 | putative lysine-specific demethylase JMJ16 |
|  | LOC101498160 | 4297558-4300243 | heavy metal-associated isoprenylated plant protein |
|  | LOC101498488 | 4305219-4314954 | type II inositol polyphosphate 5-phosphatase 15 |
|  | LOC101498836 | 4365826-4369271 | ras-related protein RABA5a, transcript variant |
|  | LOC101499979 | 4325396-4335126 | splicing factor U2AF-associated protein 2 isoform X3 |
|  | LOC101500325 | 4340795-4347673 | aspartic proteinase-like protein 2 |
|  | LOC105851868 | 4359886-4362741 | probable WRKY transcription factor 72 |
|  | LOC101500835 | 4365826-4369271 | zinc finger CCCH domain-containing protein ZFN- |
|  | LOC101501791 | 4385802-4389778 | ubiquitin carboxyl-terminal hydrolase 2-like transcript variant X2 |
|  | LOC101502115 | 4391521-4403759 | probable inactive serine/threonine-protein kinase scy1, transcript variant X1 |
|  | LOC101502957 | 4411897-4417365 | protein PELOTA 1 isoform X1 |
|  | LOC101509828 | 4425546-4428078 | subtilisin-like protease SBT1.8 |
|  | LOC101503833 | 4430186-4431718 | putative F-box protein PP2-B12 |
|  | LOC101504470 | 4444440-4446840 | F-box protein PP2 |
|  | LOC101504791 | 4449191-4452717 | probable serine/threonine-protein kinase PBL26 |
|  | LOC101505107 | 4453254-4455084 | U-box domain-containing protein 9-like |
|  | LOC101505429 | 4458864-4463569 | U11/U12 small nuclear ribonucleoprotein 48 kDa protein |
|  | LOC101506182 | 4467113-4478460 | probable UDP-N-acetylglucosamine--peptide N-acetylglucosaminyltransferase SEC isoform X3" |
|  | LOC101507066 | 4482179-4500280 | probable leucine-rich repeat receptor-like protein kinase At5g63930 |
|  | LOC101510245 | 4612369-4617652 | ER membrane protein complex |
|  | LOC101510577 | 4619725-4626876 | pentatricopeptide repeat-containing protein At1g30610, chloroplastic |
|  | LOC101511217 | 4563504-4641207 | serine/threonine-protein phosphatase PP1-like |
|  | LOC101511534 | 4660352-4661722 | phosphoenolpyruvate carboxylase kinase 1-like |
|  | LOC101511864 | 4666940-4675461 | probable serine/threonine protein phosphatase 2A regulatory subunit B''delta" |
|  | LOC101512180 | 4678261-4682700 | pentatricopeptide repeat-containing protein At1g08610 |
|  | LOC101512710 | 4684919-4689716 | RNA-binding protein 42 |
|  | LOC101514246 | 4728009-4735325 | ankyrin repeat domain-containing protein 13C |
|  | LOC101514885 | 4744759-4749434 | protein PHR1-LIKE 1 |
|  | LOC101488617 | 4770020-4772792 | cysteine-rich repeat secretory protein 3-like |
|  | LOC101489933 | 4810783-4824976 | probable inactive ATP-dependent zinc |
|  | LOC101490267 | 4825921-4829594 | leucine-rich repeat receptor-like protein kinase |
|  | LOC101490585 | 4842105-4847040 | inositol phosphorylceramide |
|  | LOC101490905 | 4854949-4859811 | bifunctional TH2 protein, mitochondrial |
|  | LOC113786369 | 4861083-4861860 | protein MAIN-LIKE 1-like involved in abiotic stress tolerance |
|  | LOC101491333 | 4881247-4892661 | myosin-11-like |
|  | LOC101492187 | 4895539-4898161 | GRF1-interacting factor 1 |
|  | LOC101492527 | 4909802-4915855 | phosphoribosylaminoimidazole carboxylase, chloroplastic" |
|  | LOC101495109 | 4948054-4951808 | protein WVD2-like 1 |
|  | LOC101495437 | 4953181-4954118 | histone H1-like |
|  | LOC101495769 | 4958118-4961262 | receptor-like protein kinase ANXUR1 |
|  | LOC101496415 | 4970499-4971639 | PRA1 family protein E-like |
|  | LOC101496747 | 4973572-4975561 | probable prefoldin subunit 4 |
|  | LOC101495647 | 8657761-8662749 | putative disease resistance RPP13-like protein2 |
|  | LOC101496750 | 8666520-8671277 | putative disease resistance RPP13-like protein2 |
|  | LOC101497058 | 8680898-8686596 | putative disease resistance RPP13-like protein2 |
|  | LOC101495649 | 8711738-8713335 | zinc finger protein ZAT5 |
|  | LOC101498163 | 8717497-8721371 | monocopper oxidase-like protein SKU5 |
|  | LOC101495972 | 8733208-8733882 | agamous-like MADS-box protein AGL80 |
|  | LOC101498490 | 8735385-8738973 | ras-related protein RABC2a-like |
|  | NAC30 | 8751448-8755936 | NAC domain containing protein 50 |
|  | NAC31 | 8769686-8772827 | NAC domain-containing protein, transcript, variant |
|  | LOC101500206 | 8782025-8784983 | 4-coumarate--CoA ligase-like 5 |
|  | LOC101500518 | 8785595-8792837 | acyl-CoA-binding domain-containing protein 4 |
|  | LOC101501276 | 8793227-8794590 | peroxidase 41-like |
|  | LOC101496309 | 8801090-8802126 | glycine-rich protein DOT1-like |
|  | LOC101501591 | 8803759-8809512 | protein BTR1 (Viruses) |
|  | LOC101496639 | 8815692-8825725 | protein MAIN-LIKE 1-like |
|  | LOC101502121 | 8825639-8826349 | histone H2B.3-like |
|  | LOC113786101 | 8849692-8850502 | protein neuralized-like |
|  | LOC101497287 | 8851708-8857173 | desmoplakin-like (Viruses) |
|  | LOC101502441 | 8858020-8860002 | leghemoglobin-like (stress) |
|  | LOC101502744 | 8862402-8864239 | leghemoglobin-like (stress) |
|  | LOC101503056 | 8864564-8868895 | probable tRNA N6-adenosine threonyl carbamoyl transferase (salt tolerance ) |
|  | LOC101503611 | 8872713-8877775 | coatomer subunit alpha-1-like (abiotic stress) |
|  | LOC101499983 | 10973204-10980122 | angio-associated migratory cell protein |
|  | LOC101500738 | 10988327-10993463 | somatic embryogenesis receptor kinase 2 |
|  | LOC101492308 | 11003296-11004792 | protein MAIN-LIKE 1-like |
|  | LOC101492641 | 11008846-11009301 | ethylene-responsive transcription factor LEP-like |
|  | LOC101492973 | 11016944-11027072 | cytochrome P450 78A7 |
|  | LOC101501048 | 11031536-11034834 | geraniol 8-hydroxylase-like |
|  | LOC101501901 | 11039669-11045950 | cleavage stimulating factor 64 |
|  | NAC33 | 11050409-11054909 | NAC domain-containing protein 16 |
|  | LOC101502862 | 11063334-11068653 | probable F-actin-capping protein subunit beta (stress) |
|  | LOC101503184 | 11071689-11079746 | tRNApseudouridine(38/39) synthase isoform X2 |
|  | LOC101504052 | 11084179-11088016 | ras-related protein Rab7-like |
|  | LOC101505767 | 11110068-11114610 | polyadenylate-binding protein 3 isoform X1 (stress) |
|  | LOC101493629 | 11133312-11134833 | F-box/kelch-repeat protein At3g23880-like |
|  | LOC101506618 | 11142940-11144628 | F-box/kelch-repeat protein At3g23880-like |
|  | LOC101506954 | 11155993-11157674 | F-box/kelch-repeat protein At3g23880-like |
|  | LOC101507586 | 11166477-11171282 | probable LRR receptor-like serine/threonine-protein kinase At1g34110 |
|  | LOC101507905 | 11184702-11187672 | ran-binding protein 1 homolog b-like" |
|  | LOC101508543 | 11204503-11214774 | type IV inositol polyphosphate 5-phosphatase |
|  | LOC101509284 | 11226944-11231434 | probable aquaporin NIP5-1 |
|  | LOC101509598 | 11231769-11236178 | probable choline kinase 1 |
|  | LOC101509931 | 11239194-11242457 | ATP synthase mitochondrial F1 complex assembly factor 1-like |
|  | LOC101510252 | 11243074-11248548 | S-adenosylmethioninecarrier 1chloroplastic/mitochondrial isoform X1 |
|  | LOC101510794 | 11252285-11259133 | carboxypeptidase SOL1 isoform X4 |
|  | GI | 11270955-11280389 | protein GIGANTEA isoform X1 (stress) |
|  | LOC101512073 | 11283475-11285849 | 40S ribosomal protein S18-like (cell death ) |
|  | LOC101512401 | 11286684-11290796 | UDP-rhamnose/UDP-galactose transporter 6 (cell death) |
|  | LOC101513918 | 11310844-11315794 | probable serine/threonine-protein kinase At1g54610 isoform X1 |
|  | LOC101514256 | 11324928-11326599 | peroxidase 12 |
|  | LOC101514570 | 11329503-11331087 | putative serine/threonine-protein kinase |
|  | LOC101514893 | 11332390-11337837 | histone-lysine N-methyltransferase ASHH1 |
|  | LOC101515218 | 11341913-11346201 | protein S-acyltransferase 8-like |
|  | LOC101488294 | 11361793-11364504 | ethylene-responsive transcription factor ERF110-like, transcript variant X1 |
|  | LOC101489407 | 11385632-11392302 | polypyrimidine tract-binding protein homolog 3 |
|  | LOC101490160 | 11395873-11399046 | 60S ribosomal protein L3-1 |
|  | LOC101490808 | 11410380-11414571 | G-box-binding factor 4-like |
|  | LOC101491663 | **11418162-11424972** | sterol 3-beta-glucosyltransferase UGT80B1 |
|  | LOC101493087 | 11440904-11442719 | 40S ribosomal protein S18-like |
|  | LOC101493413 | 11450933-11452579 | putative ABA/WDS induced protein |
|  | LOC101493944 | 11460460-11461865 | 18.1 kDa class I heat shock protein |
|  | LOC101494779 | 11489542-11493044 | tubby-like F-box protein 8 |
|  | LOC101495539 | 11551761-11554672 | trihelix transcription factor GT-2-like |
|  | LOC101495870 | 11572902-11576517 | trihelix transcription factor GT-2-like |
|  | LOC101496526 | 11620119-11629257 | ubiquitin carboxyl-terminal hydrolase MINDY-3-like isoform X2 (drought) |
|  | LOC101494895 | 11686264-11690093 | bZIP transcription factor 18-like |
|  | LOC101499479 | 11694560-11709875 | serine/threonine-protein kinase STY46-like transcript variant X2 |
|  | LOC101500740 | 11744832-11753166 | RNA-binding protein 2-like |
|  | LOC101510468 | 15700897-15705932 | elongator complex protein 2 |
|  | TRNAN-GUU | 15706047-15706120 | tRNA-Asn |
|  | LOC101511547 | 15718786-15721259 | auxin-responsive protein IAA32 isoform X1 |
|  | LOC101511875 | 15725089-15728111 | receptor protein kinase-like protein ZAR1 |
|  | LOC101513046 | 15751094-15755274 | alpha,alpha-trehalose-phosphate synthase [UDP-forming] 5 |
|  | LOC101504262 | 15765235-15766777 | reticulon-like protein B9 |
|  | LOC101514021 | 15771306-15773159 | reticulon-like protein B14 |
|  | LOC101510468 | 15700897-15705932 | elongator complex protein 2 |
|  | TRNAN-GUU | 15706047-15706120 | tRNA-Asn |
|  | LOC101511547 | 15718786-15721259 | auxin-responsive protein IAA32 isoform X1 |
|  | LOC101511875 | 15725089-15728111 | receptor protein kinase-like protein ZAR1 |
|  | LOC101513046 | 15751094-15755274 | alpha,alpha-trehalose-phosphate synthase [UDP-forming] |
|  | LOC101504262 | 15765235-15766777 | reticulon-like protein B9 |
|  | LOC101514021 | 15771306-15773159 | reticulon-like protein B14 |
|  | LOC101514681 | 15792455-15796941 | putative glutamine amidotransferase GAT1_2.1 |
|  | LOC101515334 | 15845389-15858080 | LEAF RUST 10 DISEASE-RESISTANCE LOCUS RECEPTOR-LIKE PROTEIN KINASE-like 1.2 isoform X1 |
|  | LOC101488413 | 15859023-15862082 | LEAF RUST 10 DISEASE-RESISTANCE LOCUS RECEPTOR-LIKE PROTEIN KINASE-like 1.1 |
|  | LOC101489097 | 15876277-15879178 | LEAF RUST 10 DISEASE-RESISTANCE LOCUS RECEPTOR-LIKE PROTEIN KINASE-like 2.1 |
|  | LOC101488744 | 15864571-15867755 | LEAF RUST 10 DISEASE-RESISTANCE LOCUS RECEPTOR-LIKE PROTEIN KINASE-like 1.1 |
|  | LOC101490487 | 15903990-15906721 | LEAF RUST 10 DISEASE-RESISTANCE LOCUS RECEPTOR-LIKE PROTEIN KINASE-like 1.1 |
|  | LOC101505872 | 15909661-15912720 | LEAF RUST 10 DISEASE-RESISTANCE LOCUS RECEPTOR-LIKE PROTEIN KINASE-like 1.1 |
|  | LOC101506189 | 15913623-15916285 | LEAF RUST 10 DISEASE-RESISTANCE LOCUS RECEPTOR-LIKE PROTEIN KINASE-like 2.1 |
|  | LOC105851082 | 15918167-15921503 | LEAF RUST 10 DISEASE-RESISTANCE LOCUS RECEPTOR-LIKE PROTEIN KINASE-like 1.1 |
|  | LOC101491667 | 15939455-15945306 | chaperone protein dnaJ 15 |
|  | LOC101491977 | 15956133-15957211 | zinc finger protein GIS3 |
|  | LOC101492312 | 15969265-15974226 | cysteine--tRNA ligase 2, cytoplasmic |
|  | LOC101493197 | 16026139-16027462 | protein LIFEGUARD 4-like |
|  | LOC101494157 | 16062209-16064014 | transcription factor MYB62-like |
|  | LOC101497400 | 16171717-16174257 | zinc finger protein JAGGED |
|  | LOC101507502 | 16196779-16207188 | zinc finger protein JAGGED-like |
|  | LOC101499577 | 16269591-16270806 | LOB domain-containing protein 42 |
|  | LOC101499877 | 16277341-16279374 | zinc finger protein CONSTANS-LIKE 16 |
|  | LOC101500215 | 16326545-16328463 | 3-ketoacyl-CoA synthase 6 |
|  | LOC101500525 | 16360817-16363185 | ethylene-responsive transcription factor ERF118 |
|  | LOC101501368 | 16439001-16440913 | protein trichome birefringence-like 42 |
|  | LOC101501685 | 16444054-16448514 | ubiquitin receptor RAD23c-like |
|  | LOC101502009 | 16451158-16454369 | ACD11 homolog protein |
|  | LOC101502335 | 16457073-16462232 | component 4 of pyruvate dehydrogenase complex chloroplastic |
|  | LOC101509399 | 16467637-16479142 | serine/threonine-protein kinase prpf4B |
|  | LOC101504161 | 16510453-16519310 | serine/threonine-protein phosphatase 2A 65 kDa regulatory subunit A beta isofo |
|  | LOC101505539 | 16529838-16533236 | transcription factor TGA2.3-like isoform X2 |
|  | LOC101506087 | 16535376-16538819 | CTL-like protein DDB_G0274487 isoform X1" |
|  | LOC101506410 | 16548287-16550419 | protein ASPARTIC PROTEASE IN GUARD CELL 1 protein |
|  | LOC101507592 | 16574668-16579505 | glycine-rich RNA-binding protein RZ1B isoform |
|  | LOC101509603 | 16626517-16630152 | serine/threonine protein phosphatase 2A 57 kDa regulatory subunit B' beta isoform-like |
|  | LOC101509935 | 16633005-16639925 | probable pectate lyase 18 isoform X1 |
|  | LOC101511324 | 16674028-16676318 | pentatricopeptide repeat-containing protein At5g04780, mitochondrial |
|  | LOC101511650 | 16693602-16697966 | cationic amino acid transporter 7 |
|  | LOC101510699 | 16961882-16963324 | histone-lysine N-methyltransferase 2D-like |
|  | TRNAA-UGC | 17001208-17001280 | tRNA-Ala |
|  | LOC101496204 | 17010106-17015216 | putative Myb family transcription factor |
|  | LOC101511326 | **17030797-17034638** | pentatricopeptide repeat-containing protein At5g39350 |
|  | LOC101513381 | 34797747-34800530 | pentatricopeptide repeat-containing protein At5g56310 |
|  | LOC101514030 | 34818723-34822768 | cellulose synthase-like protein D4 |
|  | LOC101514359 | 34853689-34858044 | cellulose synthase-like protein D4 |
|  | LOC101515338 | 34923386-34931500 | phytochrome-associated serine/threonine-protein phosphatase isoform X1 |
|  | LOC101488420 | 34983036-34987165 | pentatricopeptide repeat-containing protein At5g16860 |
|  | LOC101488753 | 35038105-35040047 | protein ASPARTIC PROTEASE IN GUARD CELL 2 |
|  | LOC101489300 | 35045476-35060458 | probable protein arginine N-methyltransferase 6 |
|  | LOC101489624 | 35063569-35068642 | aminoacylase-1 isoform X2 |
|  | LOC101490166 | 35096241-35097945 | nuclear transcription factor Y subunit B-1-like |
|  | LOC101495231 | 35139818-35143383 | endoglucanase 14-like |
|  | LOC101491140 | 35227689-35232653 | cyclin-J18 isoform X1 |
|  | LOC113786289 | 35308517-35310635 | importin-5-like |
|  | LOC101492317 | 35325043-35349488 | myosin-1-like |
|  | LOC101492647 | 35362864-35364718 | peroxidase 5-like |
|  | LOC101492981 | 35392824-35394758 | transcription factor TCP7-like |
|  | LOC101493313 | 35415820-35417898 | transcription factor TCP7-like |
|  | LOC101496213 | 35467370-35469507 | sugar transport protein 10-like |
|  | LOC101500649 | 35483613-35484788 | sugar transport protein 10-like |
|  | LOC101497199 | 35504645-35544726 | AUGMIN subunit 3 |
|  | LOC101497530 | 35544941-35549015 | WAT1-related protein At3g30340-like isoform X2 |
|  | LOC 101500959 | 35573884-35577363 | protein TRANSPARENT TESTA 16 |
|  | LOC 101498272 | 35630139-35633774 | elongation factor-like GTPase 1 |
|  | LOC 101498609 | 35639944-35643172 | protein MOS2 |
|  | LOC101502558 | 35646290-35649431 | lysine-specific demethylase JMJ706-like |
|  | LOC101499169 | 35651124-35653415 | chaperone protein dnaJ C76, chloroplastic |
|  | LOC 105851181 | 35664590-35666860 | pentatricopeptide repeat-containing protein At2g35030, mitochondrial isoform |
|  | LOC101503199 | 36366933-36370634 | tobamovirus multiplication protein 3-like |
|  | LOC101512527 | 36371663-36373794 | pentatricopeptide repeat-containing protein |
|  | LOC101503855 | 36378885-36380021 | putative clathrin assembly protein |
|  | LOC101504493 | 36422474-36423032 | pentatricopeptide repeat-containing protein |
|  | LOC101513382 | 36443744-36449614 | armadillo repeat-containing protein 6 |
|  | LOC101513696 | 36454008-36457326 | serine/threonine-protein kinase SRK2E |
|  | LOC101504807 | 36478391-36479803 | gibberellin 20 oxidase 2-like |
|  | LOC101514908 | 36485560-36487825 | protein DMR6-LIKE OXYGENASE 2-like |
|  | LOC101515557 | 36505452-36508141 | pollen-specific leucine-rich repeat extensin-like protein 3 |
|  | LOC101488634 | 36534022-63537537 | B3 domain-containing transcription factor VRN1-like |
|  | LOC101489625 | 36567818-36573570 | extra-large guanine nucleotide-binding protein 1-like isoform X2 |
|  | LOC101490167 | 36597635-36600880 | allene oxide synthase 3-like |
|  | LOC101490490 | 36616634-36622575 | long chain acyl-CoA synthetase 2-like |
|  | LOC101493314 | 36699274-36705411 | G-box-binding factor 1-like |
|  | LOC101493637 | 36706975-36709728 | pentatricopeptide repeat-containing protein At3g48810" |
|  | LOC101494474 | 36797018-36800288 | splicing factor U2af small subunit B |
|  | LOC105851070 | 36842319-36848604 | squamosa promoter-binding-like protein 14 |
|  | LOC101495986 | 36890489-36892842 | putative Myb family transcription factor |
|  | LOC101509613 | 37175101-37177992 | transcription factor bHLH121-like |
|  | LOC101511125 | 37258131-37270457 | dynamin-related protein 1C-like |
|  | LOC101511979 | 37272604-27280271 | microtubule-associated protein 70-2-like |
|  | LOC101513602 | 37345505-37350588 | tRNA:m(4)X modification enzyme TRM13 homolog |
|  | LOC101514465 | 37367196-37371834 | ankyrin repeat-containing protein At2g01680-like (stress) |
|  | LOC101515766 | 37422271-37424627 | nudix hydrolase 17, mitochondrial-like |
|  | LOC101488524 | 37436782-37440773 | zinc finger protein VAR3, chloroplastic |
|  | LOC101488870 | 37447500-34750732 | 1-aminocyclopropane-1-carboxylate oxidase homolog 4-like |
|  | LOC101489193 | 37452775-37458535 | zinc finger HIT domain-containing protein 2 isoform X1 |
|  | LOC101489951 | 37483259-37491952 | probable peroxygenase 4 |
|  | LOC101490284 | 37499031-37502420 | probable peroxygenase 5 |
|  | LOC101490602 | 37509529-37514903 | protein TIFY 6A isoform X1 |
|  | LOC101490924 | 37542043-37543464 | lysine-rich arabinogalactan protein 19 |
|  | LOC101491240 | 37546189-37584649 | mediator of RNA polymerase II transcription subunit 23 isoform X1 |
|  | LOC101491566 | 37607958-37612562 | glucan endo-1,3-beta-glucosidase 3-like |
|  | LOC101492547 | 37629749-37636703 | exosome complex component RRP42-like (cell death) |
|  | LOC101495006 | 37691020-37693873 | probable NAD(P)H dehydrogenase subunit CRR3, chloroplastic isoform X1 |
|  | LOC101500961 | 37803716-37806770 | nudix hydrolase 25 |
|  | LOC101502241 | 37843748-37840362 | pentatricopeptide repeat-containing protein |
|  | LOC101502756 | 37857742-37863123 | auxin efflux carrier component 4-like (stress) |
|  | LOC101503624 | 37905807-37906805 | leucine-rich repeat extensin-like protein 6 |
|  | LOC101507816 | 37930956-37932281 | F-box/kelch-repeat protein At3g23880-like |
|  | LOC101504266 | 37934930-37941552 | MLP-like protein 34 |
|  | LOC105851988 | 37996431-37998961 | protein MAIN-LIKE 1-like |
|  | LOC101508454 | 38002667-38009860 | nicotinate-nucleotide pyrophosphorylase |
|  | LOC101512309 | 38018966-38024769 | cationic amino acid transporter 1-like" |
|  | LOC101512308 | 38193290-38197689 | UDP-galactose transporter 1 isoform X1 (stress) |
|  | LOC101512638 | 38205032-38208204 | transcription factor IIIA |
|  | LOC101512948 | 28212141-28219855 | long chain acyl-CoA synthetase 9 |
|  | TRNAM-CAU | 38292482-38292566 | tRNA-Met |
|  | TRNAQ-UUG | 38294564-38294635 | tRNA-Gln |
|  | LOC101515232 | 38298212-38305954 | chlorophyllide a oxygenase, chloroplastic (necrotic cell death) |
|  | LOC101514360 | 38306741-38311545 | BTB/POZ domain-containing protein At1g21780-like |
|  | LOC101515558 | 38313849-38325388 | aldehyde dehydrogenase family 3 member H1 |
|  | LOC101515233 | 38327239-38329115 | protein MAIN-LIKE 1-like |
|  | LOC101488871 | 38355928-38366642 | protein CELLULOSE SYNTHASE INTERACTIVE 3 |
|  | LOC101489194 | 38377977-38383563 | amino acid permease 3-like |
|  | LOC101515559 | 38398843-38394739 | probable polygalacturonase At3g15720 |
|  | LOC101490056 | 38414511-38417309 | tetratricopeptide repeat protein 33 (promotion of protein–protein interactions) |
|  | LOC101490391 | 38430734-38435487 | probable receptor-like serine/threonine-protein kinase At5g57670 |
|  | LOC101489302 | 38500233-38513517 | DNA topoisomerase 2-binding protein 1-A |
|  | LOC101492431 | 38528446-38536395 | NAD kinase 2, chloroplastic-like isoform X1 |
|  | LOC101492982 | 38538260-38542119 | pentatricopeptide repeat-containing protein At5g14080 |
|  | LOC101493522 | 38548406-38550628 | probable receptor-like protein kinase At1g11050 |
|  | LOC101495232 | 38606225-38608883 | F-box protein SKP2B |
|  | LOC101495550 | 38609293-38614819 | chaperone protein dnaJ 10 |
|  | LOC101496214 | 38616073-38622836 | ras-related protein RABC1-like |
|  | LOC101497200 | 38661832-38670111 | small RNA degrading nuclease 5 isoform X1 |
|  | TRNAR-CCU | 28670209-28670281 | tRNA-Arg |
|  | LOC101497744 | 38672007-38681861 | transcriptional corepressor SEUSS |
|  | LOC101498610 | 38719693-38726950 | probable beta-1,4-xylosyltransferase IRX10 |
|  | LOC101490603 | 38735097-38737160 | F-box/LRR-repeat protein At3g48880-like |
|  | LOC101490925 | 38743691-38745540 | F-box/LRR-repeat protein At3g48880-like |
|  | LOC101498940 | 38775671-38761169 | auxin efflux carrier component 6 |
|  | LOC101499266 | 38770055-38771849 | stearoyl-[acyl-carrier-protein] 9-desaturase |
|  | LOC101491242 | 38781264-38782264 | proline-rich receptor-like protein kinase |
|  | LOC101499585 | 38783047-38788020 | putative UDP-glucuronate:xylan alpha-glucuronosyltransferase 3 |
|  | LOC101501377 | 38896605-38890734 | probable inactive receptor kinase At4g23740 |
|  | LOC101492211 | 38917738-38919654 | inorganic phosphate transporter 1-11-like |
|  | LOC101502348 | 38947561-38949414 | ethylene-responsive transcription factor CRF1-like |
|  | LOC101502656 | 38959462-38963144 | ubiquitin-conjugating enzyme E2 10 |
|  | LOC101503200 | 38964550-38968150 | SUMO-conjugating enzyme UBC9-like |
|  |  |  |  |
